# Dual-MEG interbrain synchronization during turn-taking verbal interactions between mothers and children

**DOI:** 10.1093/cercor/bhac330

**Published:** 2022-09-20

**Authors:** Jo-Fu Lotus Lin, Toshiaki Imada, Andrew N Meltzoff, Hirotoshi Hiraishi, Takashi Ikeda, Tetsuya Takahashi, Chiaki Hasegawa, Yuko Yoshimura, Mitsuru Kikuchi, Masayuki Hirata, Yoshio Minabe, Minoru Asada, Patricia K Kuhl

**Affiliations:** Institute for Learning & Brain Sciences (I-LABS), University of Washington, Portage Bay Building, University of Washington, Seattle, WA 98105, USA; Research Center for Child Mental Development, Graduate School of Medical Science, Kanazawa University, 13-1 Takaramachi, Kanazawa-City, Ishikawa-Ken 920-8640, Japan; Institute of Linguistics, National Tsing Hua University, 101, Section 2, Kuang-Fu Road, Hsinchu 300044, Taiwan; Institute for Learning & Brain Sciences (I-LABS), University of Washington, Portage Bay Building, University of Washington, Seattle, WA 98105, USA; Research Center for Child Mental Development, Graduate School of Medical Science, Kanazawa University, 13-1 Takaramachi, Kanazawa-City, Ishikawa-Ken 920-8640, Japan; Institute for Learning & Brain Sciences (I-LABS), University of Washington, Portage Bay Building, University of Washington, Seattle, WA 98105, USA; Hamamatsu University School of Medicine, 1 Chome-20-1 Handayama, Higashi Ward, Hamamatsu, Shizuoka 431-3192, Japan; Research Center for Child Mental Development, Graduate School of Medical Science, Kanazawa University, 13-1 Takaramachi, Kanazawa-City, Ishikawa-Ken 920-8640, Japan; University of Fukui, 3-9-1 Bunkyo, Fukui-shi, Fukui 910-8507, Japan; Research Center for Child Mental Development, Graduate School of Medical Science, Kanazawa University, 13-1 Takaramachi, Kanazawa-City, Ishikawa-Ken 920-8640, Japan; Research Center for Child Mental Development, Graduate School of Medical Science, Kanazawa University, 13-1 Takaramachi, Kanazawa-City, Ishikawa-Ken 920-8640, Japan; Research Center for Child Mental Development, Graduate School of Medical Science, Kanazawa University, 13-1 Takaramachi, Kanazawa-City, Ishikawa-Ken 920-8640, Japan; Department of Neurosurgery, Osaka University Medical School, 2 Chome-2 Yamadaoka, Suita, Osaka 565-0871, Japan; Research Center for Child Mental Development, Graduate School of Medical Science, Kanazawa University, 13-1 Takaramachi, Kanazawa-City, Ishikawa-Ken 920-8640, Japan; Department of Adaptive Machine Systems, Graduate School of Engineering, Osaka University, 2-1 Yamadaoka, Suita, Osaka 565-0871, Japan; Institute for Learning & Brain Sciences (I-LABS), University of Washington, Portage Bay Building, University of Washington, Seattle, WA 98105, USA

**Keywords:** MEG brain-to-brain synchrony, interbrain synchronization, turn-taking, social interaction, speech imitation

## Abstract

Verbal interaction and imitation are essential for language learning and development in young children. However, it is unclear how mother–child dyads synchronize oscillatory neural activity at the cortical level in turn-based speech interactions. Our study investigated interbrain synchrony in mother–child pairs during a turn-taking paradigm of verbal imitation. A dual-MEG (magnetoencephalography) setup was used to measure brain activity from interactive mother–child pairs simultaneously. Interpersonal neural synchronization was compared between socially interactive and noninteractive tasks (passive listening to pure tones). Interbrain networks showed increased synchronization during the socially interactive compared to noninteractive conditions in the theta and alpha bands. Enhanced interpersonal brain synchrony was observed in the right angular gyrus, right triangular, and left opercular parts of the inferior frontal gyrus. Moreover, these parietal and frontal regions appear to be the cortical hubs exhibiting a high number of interbrain connections. These cortical areas could serve as a neural marker for the interactive component in verbal social communication. The present study is the first to investigate mother–child interbrain neural synchronization during verbal social interactions using a dual-MEG setup. Our results advance our understanding of turn-taking during verbal interaction between mother–child dyads and suggest a role for social “gating” in language learning.

## Introduction

### The role of social interaction in language learning

Social interactions are considered an essential factor in different domains of learning, including neural aspects of language and social understanding (Kuhl 2007; Meltzoff et al. 2009; Kuhl 2010, 2014; Hari et al. 2015; Redcay and Schilbach 2019). With the presence of the international pandemic related to COVID-19, social distancing has been encouraged to reduce face-to-face interactions between individuals. Online teaching and learning have consequently increased in all levels of education (from elementary schools to universities). The importance and benefits of social interactions in learning have received increased attention within the USA as well as several international organizations (UNESCO, OECD, Yidan Foundation).

At the earliest ages, social interaction is known to facilitate first language acquisition (Kuhl 2010). Behavioral and electrophysiological studies have provided evidence of an essential requirement for social interaction for successful infant second language learning (Kuhl et al. 2003; Conboy et al. 2015). For adult learners of a second language, social interaction also facilitates language learning (Verga and Kotz 2013; Li and Jeong 2020).

Among different forms of social interactions, imitation is an important learning mechanism when infants observe and repeat speech samples during first language acquisition (Meltzoff 2007; Meltzoff et al. 2009). For adult learners of a second language, imitation also facilitates language learning through adjusting and correcting one’s production based on perceived speech and language models. Speech imitation is believed to be more than mechanical repetition. This is because upon hearing the speech or language samples, learners have to process the heard utterances, analyze the underlying structures, and then reproduce the utterances themselves (Jessop et al. 2007).

### Two-person neuroscience: a short history, experimental setups, and interpretations

The idea of testing 2 interactive individuals simultaneously emerged in the recent past (Hari and Kujala 2009; Hari et al. 2015). Traditionally, in most neuroimaging studies, participants are tested individually in a laboratory setting. Moreover, to exclude possible confounds, stimuli are typically simplified and well-controlled and only vary in the dimension of interest. However, because the stimuli and laboratory settings are simplified compared to what we encounter in our daily life, this possibly constrains inferences about external validity and generalizability, which has been noted in certain neuroscience publications (Hari and Kujala 2009; Levy et al. 2021). Moreover, in real-life situations such as in classrooms, learning is interactive and dynamic. Therefore, measuring 2 persons simultaneously using a more naturalistic approach will add to our current understanding of human learning. A move toward 2-person neuroscience (Hari and Kujala 2009) and experimental setups that can simultaneously scan 2 brains is required to characterize the interactions and dynamics between 2 brains during the process of learning (Dikker et al. 2017; Bevilacqua et al. 2019).

Experimental setups for testing 2 interactive individuals have moved from sequential testing, which takes only 1 system, to simultaneous testing that requires a setup that has 2 systems. The first simultaneous recordings of brain activity for 2 interacting individuals was reported by Montague et al. (2002) using a dual-fMRI (functional magnetic resonance imaging) setup . In this initial study, the term “hyperscanning” was used to refer to simultaneous recordings from more than 1 individual. Subsequently, sequential fMRI scans were utilized on 2 individuals and correlations of brain activity were analyzed (Schippers et al. 2010; Anders et al. 2011). About the same time, simultaneous recordings using dual electroencephalogram (EEG) setups started to emerge. Well-controlled studies using EEG to record brain activity from 2 individuals simultaneously have also been reported (Babiloni et al. 2006; Tognoli et al. 2007; Lindenberger et al. 2009; Astolfi et al. 2010; Dumas et al. 2010).

In the last 10 years or so, due to the technical development in dual-EEG and dual functional near-infrared spectroscopy (fNIRS) setups, rapid growth in the number of hyperscanning studies has been observed (Nozawa et al. 2016; Ayrolles et al. 2021). Using dual-EEG or dual-fNIRS setups, studies have measured brain activity from 2 interacting participants simultaneously to investigate interbrain synchronization during different types of social interactions, including synchronized finger movements (Konvalinka et al. 2014), cooperative problem solving (Jiang et al. 2015; Lu and Hao 2019), and communications (Liu et al. 2017).

Analysis approaches on intrabrain connectivity have inspired the community of 2-person neuroscience. When brain activity from single individuals is measured, statistical dependency among different neural assemblies of brain regions has been used to indicate functional connectivity (Friston 2011) or communications among different cortical areas (Fries 2005; Burgess 2013). The same analytical methods can be applied to study cross-brain interactions while 2 interactive individuals are cooperatively working on the same tasks, such as verbal communication or problem solving. These analytical methods include correlations of amplitude envelopes (Kawasaki et al. 2013; Kinreich et al. 2017; Zamm et al. 2018), coherence-based measures (Babiloni et al. 2006; Reindl et al. 2018), phase-locking values (Dumas et al. 2010; Yun et al. 2012; Pérez et al. 2017), phase lag index (Sanger et al. 2012; Ahn et al. 2018), and granger causality (Pan et al. 2018).

Evidence supporting interbrain interactions has been reported not only at the systems level in humans but also at the neuronal levels in animal studies. For example, during social interactions, interbrain correlations have been observed in pairs of interactive mice (Kingsbury et al. 2019) and bats (Zhang and Yartsev 2019). Moreover, at the neuronal level, interbrain couplings have been found in 2 neuronal populations in the prefrontal areas that have been postulated to encode social information regarding the behavior of oneself as well as that of the interactive partner (Kingsbury et al. 2019).

### Brain-to-brain synchronization during different types of social interaction

Social interaction takes a range of different forms, from synchronous finger movements, imitation of actions and speech, verbal communication, to learning in classrooms or informal settings. Because fMRI and fNIRS offer relatively good spatial resolution, researchers can capture the cortical areas involved and the patterns of interbrain interactions using dual-fMRI or dual-fNIRS recordings. With dual-fNIRS setups, imitation of finger tapping (Holper et al. 2012) and singing (Pan et al. 2018) resulted in interbrain coherence over the premotor regions and bilateral inferior frontal areas. Interbrain interactions have also been investigated with tasks involving language and verbal interaction. Using fNIRS, face-to-face dialogs enhanced cross-brain synchrony in the inferior frontal areas compared to face-to-face monologues (Jiang et al. 2012). During a cooperative word-chain game, Nozawa et al. (2016) reported enhanced coupling in the frontal areas. Hirsch et al. (2018) observed interbrain synchronization in the superior temporal and subcentral areas during object naming and description tasks. These findings reveal that face-to-face communication and cooperative interactions consistently enhance brain-to-brain synchrony in the frontal areas.

Verbal communication, like other forms of interindividual interaction, can take place in a simultaneous or turn-taking manner. Turn-taking refers to one common type of interaction when participants take turns in speaking while the other participant listens. Turn-taking based paradigms have been used to study coordinated action of speech during communication. For example, when 2 interacting participants were engaged in generating creative ideas, fNIRS measurements showed higher interbrain synchrony in the right angular gyrus in a turn-taking context compared to natural communication or an electronic brainstorming mode (Lu et al. 2020). These findings demonstrate that alternating speech tasks in healthy adults could increase between-brain connectivity between the 2 interactive individuals.

Recently, simultaneous recordings have been utilized to investigate parent–child interactions by using dual-fNIRS setups. Interbrain synchronization in the frontal areas was reported when parents and their 5-to-9-year-old children were engaged in cooperative computer games, but interbrain coupling was not observed during competitive computer games nor when adult strangers and children participated in the computer games (Reindl et al. 2018). During problem solving of a puzzle, mothers and their preschool children showed enhanced brain-to-brain synchrony in the cooperative condition relative to the condition when mothers and their children solved the puzzle individually (Nguyen et al. 2020). Specifically, higher interbrain synchrony was detected in the bilateral prefrontal and temporo-parietal regions in the cooperative than individual condition. Moreover, during free conversations between mothers and their 4–6-year-old children (Nguyen et al. 2021), turn-taking was associated with an increased level of interbrain synchrony in the temporo-parietal regions.

Similar findings have been reported in younger children. When 30-month-old children were watching an animated film together with their mothers in a dual-fNIRS setup, interbrain synchrony in the medial prefrontal region was correlated with the level of parenting stress (Azhari et al. 2019). Moreover, in a recent dual-EEG study, mothers and their 10-month-old infants were tested (Santamaria et al. 2020). Parent–infant interbrain connection was stronger within the 6–9 Hz range when the mother showed positive, compared to negative, emotion states.

Taken together, synchronous brain activity is frequently observed in the frontal and parietal regions between parents and children during social interactions. The observed mother–child interbrain synchrony could reflect mutual eye gaze, joint attention, and verbal communication. In addition, factors such as the nature of the task (being cooperative or competitive), the degree of closeness between the participants (parent–child, stranger–child), and the presence of 2 individuals in the same room, could each affect the strength of brain-to-brain coupling.

### Brain-to-brain synchronization in different frequency bands

Based on dual-EEG recordings, interbrain synchrony is often reported in the theta and alpha bands (Babiloni and Astolfi 2014), possibly reflecting a variety of cognitive processes involved. For instance, theta-band activity has been associated with attentional demands, memory formation, working memory, and cognitive loads (Klimesch 1999; Buzsaki and Moser 2013). Theta oscillations are also associated with parsing and integration of speech (Luo and Poeppel 2007; Giraud and Poeppel 2012).

Alpha oscillations have been thought to reflect attention selection, task demands, and accessing long-term memory (Klimesch 1999; Jokisch and Jensen 2007). Oscillations in the alpha band were also proposed to have functional significance in maintaining sensory information over time (VanRullen and Macdonald 2012). In a story listening task, oscillations in alpha band indicated attentional states and success of speech comprehension (Boudewyn and Carter 2018).

Beta oscillations have typically been associated with sensorimotor functions, but recent studies have pointed out its role in various cognitive functions, such as decision making, memory rehearsing, and working memory (Gross et al. 2004; Spitzer and Haegens 2017). Neural oscillations at different frequencies are known to carry different functional roles. It was proposed that oscillations in multiple frequency bands are required simultaneously for perceptual and cognitive processing (Palva and Palva 2007). For complex cognitive tasks that involve interpersonal interactions, it is likely that one cortical region in one brain interact and synchronize with the other brain in several frequency bands simultaneously.

In one of the earliest studies using dual-EEG recordings, cross-brain oscillations in the alpha and beta band were synchronized at the centro-parietal electrodes during imitation of hand gestures (Dumas et al. 2010). Similarly, alpha band interbrain synchrony also occurred at the centro-parietal electrode during motor coordination with a partner compared with a computer (Mu et al. 2016).

When tasks involved interpersonal interactions but without explicit instructions of body movement synchronization, interbrain synchronization in the theta and alpha bands was also reported. Using EEG-to-EEG recordings, larger interbrain couplings were reported when playing the prisoner’s dilemma game with a human partner in a high- compared to low-cooperative situation (Hu et al. 2018). With a human partner, theta interbrain synchrony was elevated at the fronto-central electrodes and alpha synchrony increased at centro-parietal electrodes. Moreover, when adult dyads alternated in producing alphabets (Kawasaki et al. 2013), neural synchrony was observed in the amplitude of theta and alpha ranges at the temporo-parietal electrodes.

More recently, simultaneous EEG recordings were taken with semistructured, but more naturalistic, paradigms when 2 individuals freely talked about a prespecified topic in alternating turns (Pérez et al. 2017; Pérez et al. 2019). Specifically, interbrain synchrony was observed in the central electrode sites of the speaker and frontal electrodes of the listener in the alpha band. In addition, beta band interbrain synchrony was found in the frontal electrodes of the speaker and temporal electrodes of the listener.

In general, when comparing interactive versus noninteractive conditions, studies have consistently shown elevated synchronized neural oscillations in the 2 interacting brains. In this sense, interbrain synchrony at the theta, alpha, and beta bands could be considered as neural correlates of social interaction because of its emergence across a variety of tasks, including coordinated movements, observation, imitation, empathy, and verbal interactions (Tognoli et al. 2007; Babiloni et al. 2012; Tognoli and Kelso 2015). Similar to single-brain studies, interbrain synchrony at different frequency bands could reflect different cognitive processes involved in social interactions, such as shared goals and inferring mental states of the interacting partner.

To be noted, most of the current dual-brain studies are based on a correlational approach without causal evidence (Gvirts Provolovski and Perlmutter 2021; Novembre and Iannetti 2021). To establish a causal link, brain stimulation methods could be used to manipulate interbrain synchrony and observe the subsequent social behaviors. For example, using transcranial alternating current stimulation (tACS) to stimulate the motor cortex at 20 Hz (beta) of interacting dyads, interpersonal tapping synchrony was enhanced during a finger tapping task (Novembre et al. 2017), revealing the central role of beta oscillations in supporting action initiation and alignment.

Moreover, in most dual-EEG studies, interbrain synchrony was reported at the sensor level. Sensor-level information cannot provide precise spatial information regarding the underlying neuronal sources. This is because the intervening tissues between the brain and scalp smear the potential distributions on the scalp. However, magnetic fields are less affected by the intervening tissues (Hari and Salmelin 1997). Thus, magnetoencephalography (MEG) could provide spatially more precise information regarding cortical regions showing brain-to-brain synchrony. With high temporal and spatial resolution, MEG brings a unique opportunity to study cross-brain connectivity during interpersonal interactions.

### Interbrain synchrony using MEG recordings

Compared to fMRI and fNIRS, MEG directly measures neural activity with better temporal resolution. MEG also provides better spatial resolution when compared with EEG. The advantages of using MEG for studying interbrain synchrony between interacting individuals have been highlighted by researchers (Levy et al. 2021). However, due to technical difficulties, prior to the current study, there is no previous report in the literature of simultaneous recordings of interbrain synchrony in mother–child dyads using MEG technology.

Sequential recordings with MEG systems have been used to study interbrain synchronization. To study couplings of brain activity in mother–child dyads, sequential MEG recordings of mothers and children were employed (Levy et al. 2017). Interbrain couplings were stronger in the superior temporal sulcus while 9-year-old children and their mothers were watching video clips with dialogs during positive versus conflicting interactions. The first simultaneous recordings of MEG signals from two interactive individuals were realized and validated by using 2 MEG systems located about 5 km away (Baess et al. 2012; Zhdanov et al. 2015). With cross-site MEG systems about 100 miles apart, brain activity from 2 interactive adults was recorded simultaneously during a turn-taking number-counting task (Ahn et al. 2018). Dual-MEG studies are scarce because setting up dual-MEG recordings is technically very challenging.

### The current experiment

Neural synchrony across brains may be an indicator of social interaction. However, little is known about the neural underpinnings involved, especially in terms of frequency bands and their underlying cortical areas. In the current study, and for the first time, 2 MEG systems located in the *same* magnetically shielded room (MSR) were used to capture simultaneous recordings from interactive mother–child dyads. This system has been pilot tested with mother–child pairs (Hirata et al. 2014; Hasegawa et al. 2016). Importantly, the 2 MEG systems include 1 customized child-size MEG system and 1 adult-size MEG. This setup allowed us to capture brain activity during real-time interactions between parents and their children with both participants in the same room.

The goal of our study was to identify cortical source areas underlying brain-to-brain synchrony during mother–child interactions that resemble real-life language learning scenarios. We designed a turn-taking verbal imitation task with simultaneous MEG recordings. A turn-taking verbal imitation task is more naturalistic compared to the sequential alphabet naming (Kawasaki et al. 2013) and number counting (Ahn et al. 2018) used in previous studies. While turn-taking in verbal communication indicates active involvement of conversational partners, turn-taking in the setting of language learning provides instructors and learners with immediate feedback. Upon each turn, learners can update the underlying language patterns and modify their own productions. Theories have highlighted the importance of social interactions in learning (Meltzoff et al. 2009; Kuhl 2014). Similarly, imitation seems to accelerate learning because learners observe templates (models), actively analyze and compare their own productions with the model, and then modify their own productions accordingly (Doupe and Kuhl 1999; Ahissar et al. 2001; Kuhl 2003).

Interbrain synchrony could be induced by factors other than interaction itself, for example, shared acoustic inputs and the presence of both participants in the same room (Novembre and Iannetti 2021). Therefore, we removed brain-to-speech synchronization from brain-to-brain synchronization by using partial coherence (Rosenberg et al. 1998). Moreover, a control condition was included by having mother–child dyads in the same room listening to the same auditory stimulation without social interaction.

Our study also aimed to characterize brain-to-brain synchronization in terms of the network patterns of interbrain synchrony, including the strength of interbrain neural connectivity as well as information hubs (Bullmore and Sporns 2009; Sporns 2013), which are cortical areas showing a higher number of links from one brain area to the others. In light of previous dual-brain studies, we reasoned the interactive condition would enhance cross-brain synchrony compared to the noninteractive condition. Interbrain synchrony would be enhanced in multiple frequency ranges in multiple cortical areas to support coordination of verbal behaviors in a social context. Due to the cognitive processes required in performing turn-taking verbal imitation, we expected interbrain synchrony in the theta, alpha, and low beta bands in the frontal and parietal areas, which have been implicated in the processing of speech and social information across experimental paradigms and modalities (dual-EEG, dual-fNIRS, etc.).

## Materials and methods

### Participants

Twenty-three mother–child pairs participated in this study. All were native speakers of Japanese. Five mother–child pairs were excluded because of audio recording failures or nonidentifiable audio signals. Without audio recordings, partial coherence measures cannot be performed. One pair was excluded because of no MRI acquired, and another pair withdrew from MEG scans without completion. In addition, 6 pairs with fewer than 15 accepted epochs were also excluded from further analysis due to excessive movement. In the remaining 10 pairs, the average age of the mothers was 38.7 ± 4.8 years (mean ± SD), and the average age of children was 5.3 ± 0.4 years. Prior to participation, all adult subjects provided written inform consent and all child subjects provided assent alongside parental permission. The consent, assent, and the experimental protocols were approved by the Institute of Review Board at the Kanazawa University Hospital. All mother–child pairs reported no cognitive deficits, nor speech, language, or hearing problems.

### Tasks and procedures

Each pair of the mother–child participant lay right next to each other in their respective MEG machines. Visual displays of instructions and phrases were in Japanese and shown through back-projection screens and mirrors. Details for the devices and setups can be found in Hirata et al. (2014).

**Fig. 1:**
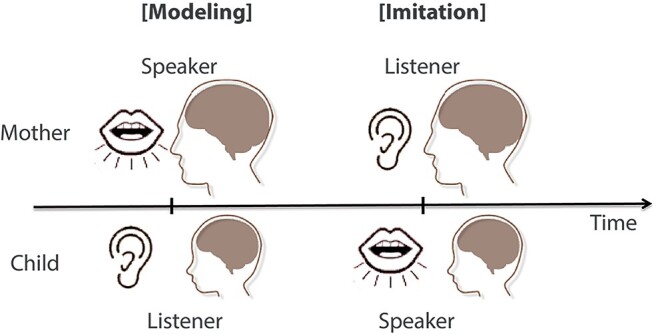
Schematic timeline of one sample trial in the interactive condition. Each trial starts with the modeling period (left) when the mother produces a Japanese phrase as the model. This is followed by the imitation period (right) when the child imitates the utterances model produced by his/her mother.

In the interactive condition, a turn-taking verbal imitation paradigm was used (Fig. 1). Mothers and their children were looking at separate screens. At the beginning of each trial, a cue (“AD” or “CD”) was displayed on the screen for the mother. For the child, a cartoon character, Pokemon, was displayed on the screen to indicate the beginning of a trial. About 2–3 s later, the modeling period started. During the modeling period, a Japanese phrase was displayed on the back-projected screen, whereas nothing was displayed on the black screen for the child. Mothers were instructed to produce the phrase using either adult-directed (AD) or child-directed (CD) intonation. CD intonation is known to be higher in overall pitch with more exaggerated intonation contours than AD intonation (Kuhl et al. 1997). The AD and CD intonations were used to add task demands and variability to pitch, but were not intended to be analyzed separately. Prior to MEG scans, instructions and examples were provided to mothers regarding AD and CD intonations addressed to children at this age. These 2 types of prosodic variations were used to enhance attention and task engagement during the interactive condition.

Then, during the imitation period, the Japanese phrase remained on the screen for the mother, whereas a black screen was displayed for the child. Children were required to imitate the mothers’ utterances, and could start their imitation when they were ready. Auditory and visual stimuli were presented using Presentation software package (Version 14.09, Neurobehavioral Systems, Inc.). The mean epoch length was 8.529 ± 1.825 s (SD), from the onset of the displayed cue sentence to the onset of experimenter evaluation. Simultaneous recordings of brain and audio signals were collected for about 7–12 min.

Faces of the conversational partner were not shown on the screen during our experiment. This is because interactive eye gaze, or eye-to-eye contact, has been found to be correlated with enhanced interbrain coherence in the frontal, temporal, and parietal regions (Hirsch et al. 2017; Noah et al. 2020). Therefore, to avoid nonverbal interactions, such as visual contacts and face-to-face interactions that might confound our main effect of speech interactions, face-to-face setups were not used in the current study.

During the interactive task, the mother–child pair listened to the same speech signals (produced either by the mother or by the child), and the child was required to respond to the mother’s utterances by imitating or repeating the intonation and words of the heard phrases. In our experiment, one experimenter stayed inside the MSR to rate the acceptability of imitated phrases. The acceptability criteria included: correct lexical items, correct word order, and correct intonation. Only trials with acceptable imitation were used in our analysis.

During the control condition, a 50 ms pure tone of 1,000 Hz was delivered through speakers to the subjects with a random interstimulus interval (ISI) between 1.5 and 1.9 s. Thus, both the mother and her child listened to the same auditory stimulation, but no social interaction was involved. During this control condition, the child was watching a silent cartoon animation of their choice, and a black screen was displayed to the mother. The mother was resting either with their eyes open or closed.

### MEG data acquisition and preprocessing

MEG signals from the mother and her child were recorded simultaneously by using 2 MEG systems: a 160-channel whole-head adult MEG system (MEGvision PQA160C; Ricoh Company, Ltd, Kanazawa, Japan) and a 151-channel child system (PQ 1151R; Ricoh Company, Ltd, Kanazawa, Japan). The 2 MEG systems were housed in the same MSR. MEG signals were recorded and digitized at 2,000 Hz with a 500 Hz analog antialiasing low-pass filter. The speech signals were recorded simultaneously with MEG data using the same MEG data acquisition hardware and settings with a sampling rate of 2,000 Hz.

MEG data preprocessing and source analysis were carried out using the Brainstorm software (Tadel et al. 2011, 2019) running on MATLAB (Mathworks, Natick, MA, USA). MEG signals were band-pass filtered between 0.5 and 200 Hz with notch filters set at 60, 120, and 180 Hz. Electrooculogram (EOG) and electrocardiogram (ECG) artifacts were marked automatically and attenuated by signal space projection (SSP) (Uusitalo and Ilmoniemi 1997). To further suppress muscle artifacts, independent component analysis (ICA) was used (Makeig et al. 1996) and muscle artifacts were manually identified and excluded. In addition, threshold-based artifact rejection was also used. To suppress artifacts related to eye movements and system instability, epochs with EOG exceeding 150 μV and MEG exceeding 3 pT were removed from offline averages. For the modeling and imitation periods, the utterance onsets and offsets were identified manually from audio recordings. For the modeling period, epochs were defined by the onset of 1.5-s long periods from the onset by the mother. For the imitation period, 1.5-s epochs were similarly defined at the onset of utterances by the children.

### MRI data acquisition and processing

Anatomical data for each subject were measured on a 1.5 T MR scanner (Signa Excite, GE Medical Systems Ltd, Milwaukee, WI, USA). Three-dimensional *T*_1_-weighted structural images were obtained from adults and children.

Prior to the MEG recordings, three anatomical landmarks (nasion, left preauricular point, and the right preauricular point) and the head shapes were digitized for later coregistration between the MEG channels and the anatomical MRI.

### MEG source analysis

Noise covariance matrices were computed using the 100 ms prestimulus baseline of each epoch. The forward models were calculated based on the above *T*_1_-weighted MRI data using the overlapping spheres approach (Huang et al. 1999) implemented in Brainstorm. Based on anatomical images of each subject, the cortical surface was tessellated into 15,000 vertices along the gray/white matter interface. MEG sources at these vertices with loose orientation constraints were estimated using weighted minimum norm estimates (Baillet et al. 2001) at each time point to map the measured MEG data onto cortical surfaces. Then, the source activity was normalized by sLORETA (Pascual-Marqui 2002). Source estimates from each individual subject were morphed to an average brain provided by FreeSurfer (subject “fsaverage”) (Dale et al. 1999; Fischl et al. 1999). Based on the Destrieux atlas (Destrieux et al. 2010) provided by FreeSurfer, source activity from 88 anatomically-defined region of interests (ROIs) were extracted using the first principal component across all vertices within a given label (see Supplementary Table S1 for a list of the anatomical labels and their corresponding numbers).

### Interbrain synchronization

To examine brain-to-brain synchronization between the mother–child pairs, coherence was computed between the source activity from the mother and her child. The source activity was obtained from the first principal components extracted from each ROI. Coherence was computed for all combinations of brain areas (ROIs) between the mother’s brain and the child’s brain. Coherence is a measure of correlation in the frequency domain where 0 represents no dependency between 2 source signals and 1 represents perfect dependency and complete similarity. Similarity between neural signals is interpreted as neural synchrony between 2 brains or 2 cortical areas. Coherence of brain signals from the mothers and their children were computed using the multitaper spectral estimation method or discrete prolate spheroidal sequence (DPSS) tapers (Slepian 1978; Thomson 1982) for each single-epoch waveform (Mitra and Pesaran 1999), as implemented in MNE-python (Gramfort et al. 2013, 2014). The same multitaper method was used to calculate coherence between the recorded speech signals and brain signals. To be noted, coherence measures similarity between 2 signals in terms of both power and phase. Coherence was used here to measure similarity of cognitive states between 2 individuals (Ayrolles et al. 2021; Turk et al. 2022).

In the current study, the source activity was analyzed in frequency bands of theta (4–7 Hz), alpha (8–12 Hz), and low beta (13–17 Hz). The low beta band has been defined differently across studies, either from 13 to 15 Hz (Konvalinka et al. 2014) or from 9 to 17 Hz (Mukherjee et al. 2019). Because speech production was required in our verbal imitation tasks, MEG signals were likely contaminated by muscle artifacts. As speech-related muscle artifacts are mostly observed at high frequencies above 20 Hz (Muthukumaraswamy 2013), focusing on the low beta band helped avoid contamination by high-frequency muscle artifacts.

### Partial coherence analysis

In our study, because mother–child dyads lay adjacently in the same MSR, interlocutors naturally heard the same utterances and thus receive similar auditory stimuli during verbal communication. Thus, the observed interbrain synchronization between interlocutors could be driven by either the low-level processing of shared sensory inputs or the higher-level effects of social interaction itself.

In the interbrain synchronization literature, the challenge of cross-brain synchronization being related to identical sensory inputs has been noted (Novembre and Iannetti 2021). To help disentangle the effect of acoustic signals and social interaction, the coherence between brain activity and the speech signals was also calculated. Then, we used partial coherence (Rosenberg et al. 1998) to identify interbrain synchronization in the mother–child pairs when the common influence of acoustic signals was removed to focus on the brain-to-brain neural synchrony directly caused by social interaction. In an MEG study using single-subject approach (Park et al. 2016), partial coherence was used to separate brain-to-lip and brain-to-audio coherence. Researchers have also suggested to use partial correlation or multiple linear regression to disentangle synchronized brain activity and identical sensory stimuli (Pérez et al. 2017; Czeszumski et al. 2020). Specifically, for each epoch, coherence was calculated for all combinations of the low-passed speech signal envelopes and the brain activity extracted from the ROIs. Then, interbrain synchronization was calculated using partial coherence to remove the effect of brain-to-speech synchronization. Thereafter, partial coherence values were converted to *z*-values using Fisher’s *z*-transformation before further statistical analysis.

### Statistical analysis

We hypothesized that larger coherence values in the theta, alpha, and low beta bands would be observed when mother–child interaction was required, compared with the condition when no interaction was involved. To test whether brain-to-brain synchronization was enhanced during the interactive in contrast to the noninteractive condition, brain-to-brain partial coherence values were compared across the interactive and control (noninteractive) conditions using paired *t*-tests for each of the 88-by-88 ROI–ROI connectivity values. This contrast also helped control for shared auditory inputs driving the interbrain synchronization. To test whether the observed differences between conditions are above chance level, we used a permutation test to randomly shuffle condition labels within each pair over 1,000 iterations. The false discovery rate (FDR) method (Benjamini and Hochberg 1995) was used to correct for multiple comparisons.

**Fig. 2:**
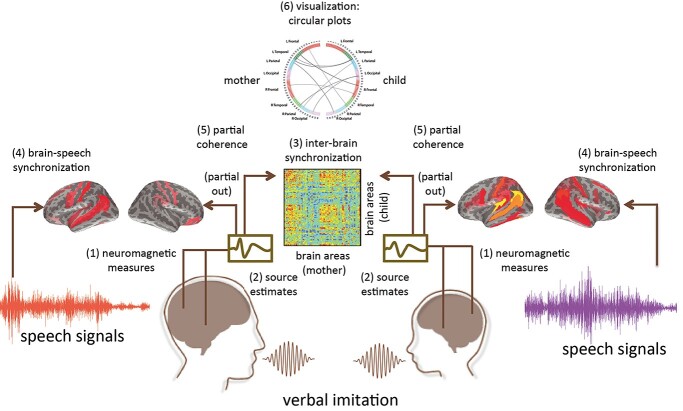
Analysis steps for calculating interbrain synchronization. This schematic diagram shows our analysis pipeline for one single pair of subjects. (1) Neuromagnetic signals were recorded simultaneously from two interactive individuals. (2) Source activity was estimated. (3) Brain-to-brain synchronization was calculated between 2 source time-series signals from the interacting individuals (e.g. the mother–child pair). (4) Brain-to-speech synchronization between the source activity and the speech signals was also computed. (5) Then, partial coherence was calculated to remove the contributions of shared acoustic signals on interbrain synchronization. (6) Results of interpersonal brain synchronization were visualized using circular plots.

Significant differences in partial coherence between the interactive and noninteractive conditions were displayed on the cortical surfaces of inflated brains. Interbrain connections were also visualized using circular plots. Figure 2 illustrates the analysis pipeline used in the present study.

### Hub analysis

Following the partial coherence analysis, we quantified the “hubness” of each ROI by calculating the “degree” of each ROI (Sporns 2018). The degree measure is a metric in graph theory (Bullmore and Sporns 2009; Sporns 2013) and calculated the interbrain density as the ratio of significant interbrain connections to the total number of interbrain connections. This was done by thresholding the partial coherence matrix using a proportional threshold by taking the top 15% of the strongest connections in the network (Garrison et al. 2015; Gerloff et al. 2022). These interbrain connections were also significant with *P* < 0.005 (FDR-corrected). Then, the degree measure was obtained by computing the fraction of significant interbrain connections to all possible connections to that ROI (out of 88).

## Results

### Behavioral measures

During MEG recordings, 1 experimenter stayed in the MSR to rate the accuracy of imitations. Only accurately imitated epochs were kept for further analysis. A total of 50 trials were tested. On average, 36 ± 4 (mean ± SD) of the total trials were evaluated as correct imitations. The mean imitation accuracy was 72.0 ± 8.0%, which is high. On average, the gap between the offset of a mother’s utterances and the onset of her child’s imitation was 1.265 ± 0.465 s (mean ± SD).

### Brain measures

The major research question of the present study was to investigate the effect of turn-taking verbal interactions on interbrain synchrony between mother–child dyads. Across our frequency bands of interest (theta, alpha, and low beta), statistically significant interbrain coherence was obtained in the same ROI pairs (or, homologous areas; e.g. the right inferior frontal gyrus of the mother and the right inferior frontal gyrus of the child) as well as different ROI pairs (or, nonhomologous areas; e.g. the right inferior frontal gyrus of the mother and the left transverse temporal sulcus of the child).

In our analysis, partial coherence measures were first calculated for the interactive and noninteractive conditions separately within 3 frequency bands (Supplementary Fig. S3). The interactive and noninteractive conditions differ in 2 aspects, low-level processing (e.g. language versus simple tones) and high-level processing (e.g. with and without interpersonal interactions). To identify interbrain synchrony associated with these 2 aspects, partial coherence values were compared between the interactive and noninteractive conditions.

**Fig. 3:**
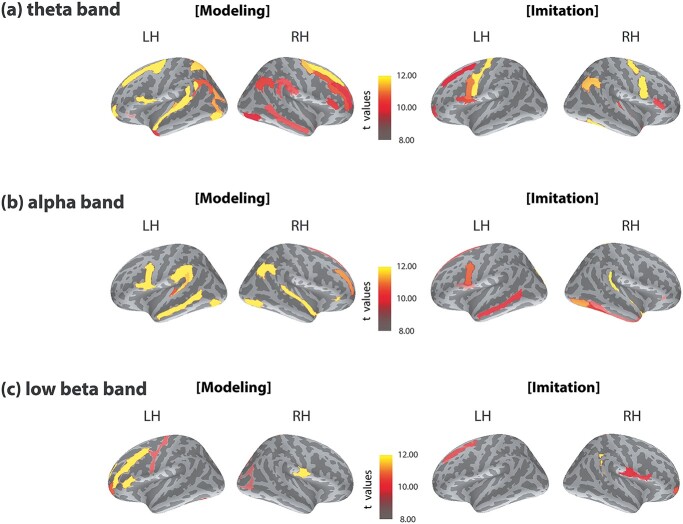
Interbrain synchrony of homologous areas. Statistical comparisons of socially interactive > noninteractive condition during the modeling period (left) and the imitation period (right) in the (a) theta band, (b) alpha band, and (c) low beta band. Interbrain partial coherence was calculated by removing the contributions from auditory signals. Cortical areas showing significant differences in interbrain synchronization are shown on the cortical surfaces (*P* < 0.005, FDR corrected). Only ROI pairs constituting the same region of the mothers and the children are displayed (e.g. the right angular gyrus of the mother and the right angular gyrus of the child) in this figure. See Supplementary Table S2 for the list of brain areas showing significant differences.

In addition, brain-to-brain partial coherence was calculated after removing the contributions of shared speech signals and pure tone acoustic signals. Thus, our interbrain partial coherence reveals interbrain synchronization that is not explained by acoustic signals. Compared to the original coherence analysis (Supplementary Fig. S1), partial coherence analysis showed fewer cortical regions with a reduced level of coherence (Fig. 3). For example, during the modeling phase, theta interbrain coherence in the right angular gyrus of the mother and child was reduced from *t* = 18.86 (original coherence) to *t* = 10.24 (partial coherence) (FDR corrected; *P* < 0.005). During the imitation phase, theta interbrain coherence in the left pars opercularis of the inferior frontal gyrus decreased from *t* = 12.21 (original coherence) to 10.23 (partial coherence) (FDR corrected; *P* < 0.005) (Supplementary Fig. S2).

### Interbrain synchronization: homologous areas

Partial coherence values were significantly higher in the interactive than the noninteractive condition during both the modeling and imitation phases. Specifically, increased interbrain synchronization was found in homologous areas of the mothers and the children. All brain areas reported below showed significant differences between the interactive and noninteractive conditions (FDR corrected; *P* < 0.005) (see Supplementary Table S2 for a list of cortical regions with significance differences).

#### Theta band

During the modeling phase (Fig. 3a, left), enhanced interbrain synchrony was found in the left temporal areas, including the planum temporale, temporal pole, and transverse temporal sulcus. Increased neural synchrony between mothers and children was also detected in the left frontal and parietal areas, such as the superior part of the precentral sulcus, pars opercularis of the inferior frontal gyrus, superior frontal sulcus, angular gyrus, and superior parietal lobule. In the right hemisphere, stronger brain-to-brain synchronization was observed in the temporal, frontal, and parietal regions, including the middle temporal gyrus, pars triangularis of the inferior frontal gyrus, superior part of the precentral sulcus, middle frontal gyrus and sulcus, supramarginal gyrus, and angular gyrus.

During the imitation phase (Fig. 3a, right), neural synchrony between brains increased significantly during the interactive condition in the bilateral frontal regions, such as the left precentral gyrus, left pars opercularis of the inferior frontal gyrus, left superior frontal sulcus, right pars triangular of the inferior frontal gyrus, and left and right inferior part of the precentral sulcus. In the right hemisphere, higher interbrain synchrony was detected in the temporal and parietal areas, including the angular gyrus and transverse temporal sulcus.

Overall, across the modeling and imitation periods, increased theta interbrain synchrony was found in the left pars opercularis of the inferior frontal gyrus, right pars triangularis of the inferior frontal gyrus, and right angular gyrus. The interpersonal neural synchrony was observed among the same cortical regions of the interactive mother–child pairs, indicating that the same brain regions were synchronized between the interactive individuals.

#### Alpha band

During the modeling period (Fig. 3b, left), the interactive condition induced a significant increase in interbrain synchrony in the frontal, temporal, and parietal areas. For example, increased interbrain connections were detected in the left inferior part of the precentral sulcus, pars opercularis of the inferior frontal gyrus, planum temporale, transverse temporal sulcus, middle temporal gyrus, and supramarginal gyrus. In the right hemisphere, significantly greater interbrain synchrony was detected in the middle frontal sulcus, superior frontal gyrus, superior temporal gyrus, and the angular gyrus.

During the imitation period (Fig. 3b, right), the interactive and noninteractive condition differed in interbrain synchrony in the frontal and temporal areas. Higher interbrain connections were observed in the left inferior part of the precentral sulcus, pars opercularis of the inferior frontal gyrus, superior frontal gyrus, and middle temporal gyrus in the left hemisphere. In the right hemisphere, stronger interbrain synchrony was found in the pars orbitalis of the inferior frontal gyrus, planum temporale, temporal pole, calcarine fissure, and lateral occipital temporal sulcus.

In summary, stronger alpha interbrain synchrony appeared to be in the left pars opercularis of the inferior frontal gyrus, inferior part of the precentral sulcus, and left middle temporal gyrus the across the modeling and imitation periods.

#### Low beta band

Interbrain synchrony increased significantly during the modeling period (Fig. 3c, left) of the interactive condition in the frontal regions bilaterally, including the left pars triangularis of the inferior frontal gyrus, left middle frontal gyrus, left precentral gyrus, and left fusiform gyrus. In the right hemisphere, higher interbrain connections were observed in the paracentral lobule and sulcus, subcentral gyrus and sulcus, cuneus, superior occipital gyrus, and precuneus.

During the imitation period (Fig. 3c, right), interpersonal neural synchrony was stronger in the bilateral frontal areas, including the left superior frontal sulcus, the right precuneus, right cuneus, and right pars opercularis of the inferior frontal gyrus.

To summarize, across the modeling and imitation periods, interbrain synchrony increased in the right paracentral lobule and sulcus in the low beta band.

### Interbrain synchronization: nonhomologous areas

**Fig. 4:**
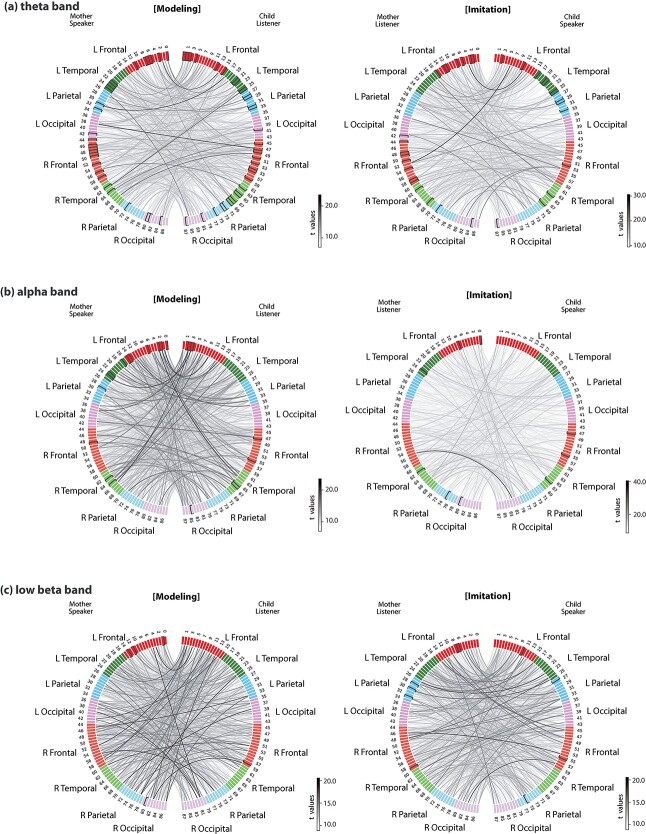
Circular plots showing significant interbrain synchronization. Lines between cortical regions indicate significant connections between the brains of the mothers and children from statistical comparisons of socially interactive > noninteractive condition during the modeling period (left) and the imitation period (right) in the (a) theta, (b) alpha, and (c) low beta frequency bands. The connected lines are color-coded in gray by paired *t*-test statistics (FDR-corrected, *P* < 0.005): Darker lines denote higher *t*-values. Each line represents a connection between the mothers (left side of the circle) and children (right side of the circle). Only the top 15% of connections are displayed. In each of the circular plots, the left side shows each cortical region of the mothers analyzed, whereas the right side represents regions from the children. Cortical regions are organized and color-coded based on their locations (e.g. red: LH frontal, green: LH temporal, blue: LH parietal, purple: LH occipital, orange: RH frontal, light green: RH temporal, light blue: RH parietal, light purple: RH occipital). Individual labels are enumerated in supplementary information (Supplementary Table S1). See Supplementary Table S3 for a list of significant interbrain connections.

Enhanced brain-to-brain connectivity was also observed in nonhomologous cortical regions of the mother–child pairs. To visualize significant interbrain connectivity, we used circular plots with ROIs from the mother on the left and the ROIs from the children on the right (Fig. 4). Interbrain connections among the top 15% of partial coherence values were displayed. All these interbrain connections were significant at the *P* < 0.005 level (FDR corrected) (see Supplementary Table S3 for detailed and complete information).

#### Theta band

During the modeling phase (Fig. 4a, left), prominent interbrain connections were found between the left frontal regions of the mother and the left frontal and bilateral temporal regions of the child. For example, the left pars opercularis of the inferior frontal gyrus of the mother was connected with the left inferior part of the precentral sulcus of the child (M2 ↔ C14; *t* = 23.01), and the left superior frontal gyrus of the mother was connected with the left planum temporale of the child (M0 ↔ C19; *t* = 19.14). Across-hemispheric synchrony was also found in the left triangular part of the inferior frontal gyrus of the mother and the right transverse temporal sulcus of the child (M4 ↔ C68; *t* = 17.43). Within the right hemisphere, the right frontal areas of the mothers presented connections with the right frontal and temporal regions of the children. In addition, the right temporal areas of the mothers showed connections with the right frontal and left temporal areas of the children. For instance, the middle temporal gyrus of the mother was connected to the middle frontal gyrus of the child (M64 ↔ C45; *t* = 20.50).

During the imitation phase (Fig. 4a, right), increased interbrain synchrony was detected between the left frontal and temporal regions of the mothers and the left frontal and right temporal regions of the children. For instance, the left middle temporal gyrus of the mother showed connections with the left paracentral lobule and sulcus of the child (M20 ↔ C7; *t* = 18.63). Within the right hemisphere, the right frontal areas of the mother presented connections with left frontal and parietal areas of the children. The right temporal regions of the mothers connected with the right frontal and temporal regions of the children. Specifically, the right transverse temporal sulcus of the mother was connected with the right inferior part of the precentral sulcus of the child (M68 ↔ C58; *t* = 22.70), the left superior temporal gyrus of the mother was connected with the right transverse temporal gyrus of the child (M17 ↔ C60; *t* = 20.94).

#### Alpha band

During the modeling period (Fig. 4b, left), increased interbrain connections were observed in the interactive in contrast to the noninteraction conditions. The left frontal areas of the mother showed increased connections with the left frontal, and right temporal areas of the children. The left temporal area of the mother had strong connection with the left frontal area of the children. Within the right hemisphere, the frontal regions of the mothers presented connections with the right temporal areas of the children. The right temporal regions of the mother had connections with the left frontal and temporal regions of the children.

During the imitation period (Fig. 4b, right), interbrain connections were observed between the left frontal areas of the mother and the right temporal and parietal areas of the children. Connections were also found between the left temporal regions of the mothers and the left parietal regions of the children. In the right hemisphere, the right frontal and temporal areas of the mothers showed connections with the right occipital and left frontal areas of the children.

#### Low beta band

The analysis revealed interbrain connections between the left frontal areas of the mother and the left frontal and right occipital areas of the children during the modeling period (Fig. 4c, left). Interbrain connections were also found in the right frontal areas of the mother and right parietal areas of the children.

During the imitation period (Fig. 4c, right), left frontal regions of the mother presented connections with the right frontal areas of the children. The left parietal regions showed connections with the left temporal regions of the children. In addition, the right frontal areas had connections with the right frontal and left temporal areas of the children.

### Interbrain synchronization: information hubs

**Fig. 5:**
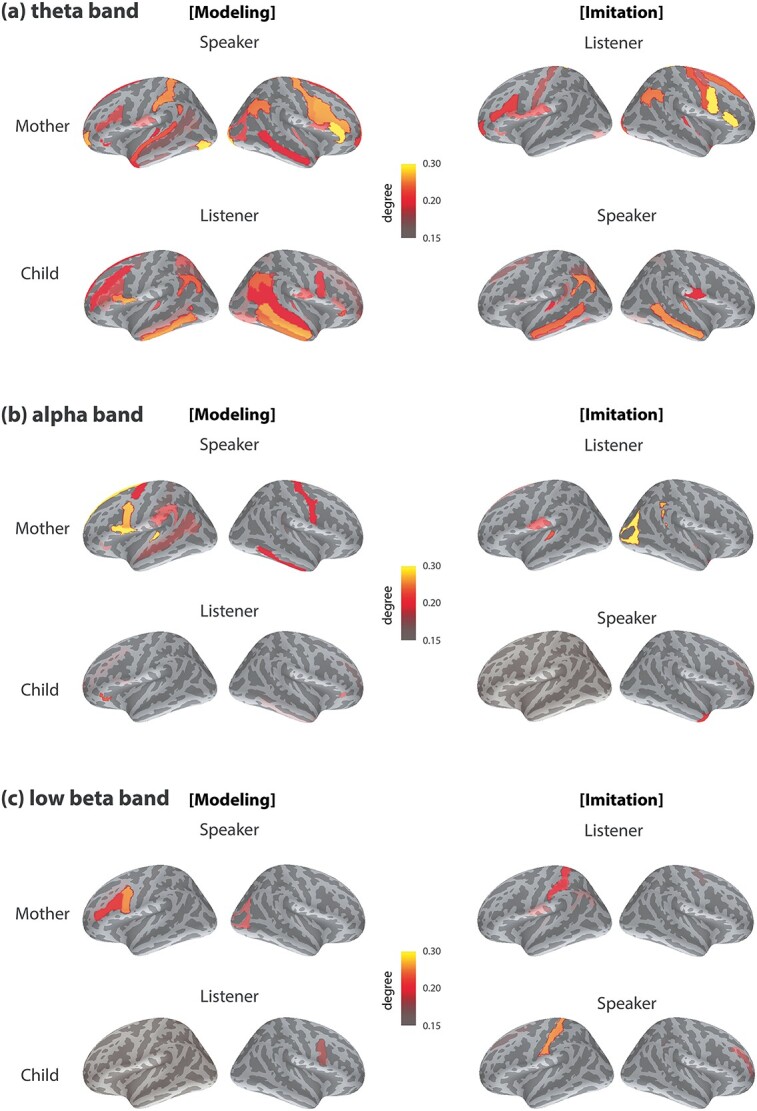
Cortical hubs on the cortical surfaces. (Left) Cortical areas showing a relative high measure of degree, or a relatively high number of interbrain connections, in the (a) theta, (b) alpha, and (c) low beta bands are represented on the cortical surfaces. Nodes for network hubs are marked by a black border color in Figure 4. See Supplementary Table S4 for the list of the cortical hubs.

To find out important cortical hot spots, we further calculated the degree measure of each ROI, which is the fraction of thresholded connections (proportional scaling) to the total possible connections to that ROI. Figure 5 illustrates the top 15% strongest connections showing significance (*P* < 0.005; FDR corrected). A complete list of frequency-specific cortical hubs can be found in Supplementary Table S4.

#### Theta band

During the modeling phase, the mothers produced Japanese phrases with different prosodies while their children listened to the utterances. During this period, several cortical hubs were observed in the mothers’ brains that showed a high number of interbrain connections with the children’s brains. Common cortical hubs were found in both mothers and children, such as the right angular gyrus (M72, C72) and right pars triangularis of the inferior frontal gyrus (M48, C48) (Fig. 5a, left).

During the imitation phase, children repeated the utterance produced by the mothers while their mothers listened to the imitated phrases. During this period, one common network hub was observed in mothers and children: the left pars opercularis of the inferior frontal gyrus (M2, C2) (Fig. 5a, right).

Overall, in the mothers, cortical hubs that were found common to both the modeling and imitation phases included the right pars triangularis of the inferior frontal gyrus (M48) and right angular gyrus (M72). In the children, network hubs emerged during both the modeling and imitation phases were the left pars opercularis of the inferior frontal gyrus (C2) and left angular gyrus (C28).

#### Alpha band

During the modeling period (Fig. 5b, left), one cortical hub was identified both in the mothers and children: the left pars opercularis of the inferior frontal gyrus (M2, C2). During the imitation period (Fig. 5b, right), no common cortical hubs were observed.

In summary, in the mothers, cortical hubs common to both the modeling and imitation periods was the left transverse temporal sulcus (M24). In the children, common hub locations found across the modeling and imitation periods were in the right pars orbitalis (C47) and the right middle frontal sulcus (C55).

#### Low beta band

No cortical hubs were observed between the mothers and children from either the modeling (Fig. 5c, left) or the imitation period (Fig. 5c, right). In addition, no common hub areas were found during the modeling and imitation periods in either the mothers or children.

## Discussion

The present study measured brain signals simultaneously from interacting mother–child pairs utilizing a unique dual-MEG setup for the first time. We studied cross-brain interactions in mother–child dyads who were engaged in a social turn-taking verbal imitation task and a passive listening task.

At the behavioral level, the turn-taking time observed during the interactive condition inside the MEG scanning room was slightly longer than those previously reported. From behavioral studies on turning taking in conversations, the average gap observed between turns is about 250 ms across languages (Stivers et al. 2009). The transition gaps were primarily between 200 and 500 ms (Wilson and Wilson 2005; Levinson and Torreira 2015). A longer turn-taking time observed in our study could suggest that our imitation paradigm in a laboratory setting still differs somewhat from spontaneous conversations in natural settings.

At the cortical level, significantly higher brain-to-brain synchrony was detected during the interactive imitation condition as compared to the noninteraction listening condition. Increased interbrain synchrony was observed in cortical hubs in the interactive mother–child dyads in right parietal and bilateral frontal areas (Figs 3 and 5). This increased synchrony was specific for verbal communication when nonverbal cues, such as facial expressions, were avoided and acoustic modulations were partialled out. These findings provide neural evidence that social interaction enhances interpersonal neural synchrony.

### The role of shared acoustic inputs in interbrain synchrony

When interpreting interbrain synchronization, 2 perspectives have been proposed. First, the “epiphenomenon” perspective states that interbrain synchrony is purely caused by the common external stimuli or sensory inputs, and interbrain synchronization occurs even without interpersonal interactions (Burgess 2013). Alternatively, a mechanistic view suggests that the observed interbrain synchrony reflects neural mechanisms underlying social interaction, and interbrain synchrony facilitates social behaviors (Gvirts Provolovski and Perlmutter 2021; Novembre and Iannetti 2021). On this view, interbrain synchrony is independent of shared or common external influences (Wass et al. 2020).

According to a recent model proposed by Hoehl et al. (2021), temporal regularities in the external signals could initiate brain responses in a bottom-up manner and social–emotional factors could modulate alignment of brain activity in a top-down manner. In the present study, when mother–child pairs were involved in turn-taking verbal imitation tasks in a socially interactive setting, identical acoustic signals of speech as well as turn-taking interactions could both contribute to the observed interbrain synchronization. Indeed, our original coherence measures include both the epiphenomenal and mechanistic effects. Hence, in the current study, we adopted an analytic approach (i.e. partial coherence) that removed the bottom-up effects of identical acoustic inputs. Therefore, the brain-to-brain connectivity measured by partial coherence in the present study was not due to shared external stimuli.

Before removing the common influence of shared speech, significant interbrain synchrony was found in our original coherence analysis (Supplementary Fig. S1), indicating the role of external and shared speech signals in synchronizing interpersonal neural activity. After removing the common influence of shared speech, significant interbrain synchrony remained (Fig. 3). Moreover, we observed overlapping areas from the original coherence and partial coherence measures. Comparing results before and after taking into account the presence of shared acoustic inputs, higher coherence values were observed in the original coherence analysis (Supplementary Fig. S2). Therefore, after removal of the effects from identical sensory stimulation, the remaining synchronous oscillations are consistent with the idea that these oscillations reflect a neural mechanism underlying social interactions.

As suggested in previous studies, the observed interbrain synchrony in the present study could arise from both low-level sensory (epiphenomenal) and high-level communicative goals or shared processes (mechanistic) (Pérez et al. 2017; Kelsen et al. 2020; Gugnowska et al. 2022; Schaefer and Bittmann 2022). For example, social signals, such as vocalization and speech, could trigger and temporally enhance interpersonal neural synchrony (Wass et al. 2020). Thus, we speculate that speech signals could bring the two interacting brains into synchronization, creating an interbrain link. This synchronized interbrain functional connection is further enhanced by the intention to coordinate behaviors and/or share social goals, which could possibly facilitate communications across individuals during social interactions.

Brain stimulation can also be used to tease apart the epiphenomenal and mechanistic effects (Novembre and Iannetti 2021). Interestingly, to investigate whether externally stimulated interbrain synchrony could promote learning of new songs between the instructor–learner pairs, a dual brain stimulation setup with transcranial alternating currents stimulators (tACS) was used (Pan et al. 2021). When two brains were stimulated at the theta frequency range with tACS, brain activity between the instructor and learner became synchronized. As a result, learning outcomes of new songs were enhanced. Likewise, findings from our study suggest that the shared auditory stimulation of speech serves a role of stimulating and synchronizing brain activity from the mother–child pairs. Thus, interpersonal neural synchrony can be induced by common inputs in the external environments as well as brain stimulation (Novembre and Iannetti 2021). In contrast to brain stimulations, shared acoustic signals could be used noninvasively and potentially boost learning outcomes.

### Interbrain coherence in the right angular gyrus

The present study showed that speech interactions in a turn-taking based paradigm increased interbrain synchrony in the right angular gyrus of the mothers and children in the theta band across the modeling and imitation stages. Moreover, when calculating the node degree, the right angular gyrus appeared to be one of the common hubs of interbrain connectivity across mothers and children during the modeling stage in the theta band. During the imitation stage, the right angular gyrus was also identified as a cortical hub in the mothers.

In our interactive verbal imitation task, focused attention is required to track the speech of another person. In addition, shared intentions, mutual understanding, and coordinated verbal behaviors are required to successfully perform the task at hand. Therefore, we attribute the synchronized theta oscillations in the right angular gyrus to coordinated speech and social interactions. Anatomically, the angular gyrus is part of the temporoparietal junction (TPJ) (Carter and Huettel 2013). When considering its functional roles, the TPJ plays a central role in attentional control as well as social cognition (Decety and Lamm 2007; Kubit and Jack 2013; Schuwerk et al. 2017; Seghier 2022).

With the single-person approach, the TPJ is typically recruited when tasks demand inferring the mental state or intentions of others (Saxe et al. 2006; Lamm et al. 2010; Frith and Frith 2011; Morelli et al. 2014). Interestingly, the right TPJ increased activation during face-to-face live interactions compared to recorded interactions (Redcay et al. 2010). The causal relationship between right TPJ has also been verified. While stimulating the right TPJ with transcranial direct current stimulation, participants showed improved performance in tasks involving imitation and perspective taking (Santiesteban et al. 2012).

In studies using the 2-person approach, activation of the right TPJ by the presence of another live person has been further validated. Using dual-fMRI recordings, coupling of fMRI responses was observed in the right TPJ during joint attention (Bilek et al. 2015), joint force production (Abe et al. 2019), and joint attention of eye gaze (Goelman et al. 2019). More recently, a 3-fMRI setup was used to investigate interpersonal neural synchrony in a multibrain framework. When tested on a verb drawing task, triads showed increased brain synchronization in the right TPJ during the collaborative condition (Xie et al. 2020). Together, fMRI studies on single person as well as 2 interactive individuals indicate that the right TPJ could be activated when a live person is absent but the task demands inferring the mental states of others, or when a live person is present and the task involves cooperative interactions.

Dual-fNIRS recordings have also been used to investigate cross-brain networks underlying cooperative and collaborative behaviors. A higher degree of cross-brain synchronization was observed in the right TPJ when pairs of individuals were in face-to-face settings and engaged in an adapted version of an ultimatum game (Tang et al. 2016) and generating creative ideas (Lu et al. 2019; Lu et al. 2020). Increased interbrain synchrony in the right TPJ and prefrontal areas has been reported during a face-to-face deception task (Chen et al. 2020) and during trust development in an economic exchange game (Cheng et al. 2022). These findings provide supporting evidence that synchronized activity in the right parietal region is associated with social closeness, shared goals, and understanding of the other individual.

In dual-EEG studies, oscillatory activity has shown interbrain synchrony in the alpha band at the parietal electrodes. Using dual-brain EEG recordings, neural oscillations in the alpha and beta bands showed greater interbrain synchrony during imitation of hand movements at electrodes located above the centroparietal regions (Dumas et al. 2010). Moreover, participants showed greater interbrain synchronization in the alpha band at the right temporoparietal electrodes during face-to-face compared to face-blocked Prisoner’s Dilemma Game (Jahng et al. 2017). These findings highlight the importance of the alpha band in coordinated and collaborative tasks.

Interbrain synchronization in the theta band has also been reported at the parietal electrodes using turn-taking paradigms with dual-EEG setups. When pairs of participants were asked to name alphabets sequentially in a turn-based manner, interactive individuals showed brain-to-brain synchronization in the theta and alpha bands at the temporal and parietal electrodes (Kawasaki et al. 2013). The theta and alpha synchronization could also reflect the effects of working memory, which was required in order to successfully produce alphabets sequentially in a turn-taking manner with partners. In the present study, interbrain communication was observed in the right parietal area in the theta frequency band. In terms of frequency ranges, differences in tasks and contexts could lead to slightly different synchronization patterns across the 2 interacting brains. For example, our turn-taking verbal imitation task was more naturalistic and required more complex linguistic structures compared to the turn-taking paradigms used in previous studies (Kawasaki et al. 2013; Ahn et al. 2018).

However, brain-to-brain coherence in the right parietal areas could simply reflect an effect of eye-to-eye contact. Increased cross-brain synchrony in the right angular gyrus was also reported during eye-to-eye contact compared to eye gaze on a face video (Hirsch et al. 2017; Noah et al. 2020). Therefore, it is important to ensure interpersonal neural synchrony observed is not driven by eye-to-eye contact. In the present study, eye contact was not possible between mother–child pairs. By eliminating possible confounds of eye contact and face processing, our study revealed enhanced interbrain synchronization in TPJ during the interactive compared to noninteractive task.

To be noted, although mutual gaze was not possible in our study, visual information was not identical across conditions. In the interactive condition, the mother was receiving more visual inputs for a longer period of time (i.e. the Japanese phrases stayed on the screen for the modeling as well as imitation period). In the noninteractive condition, the child was receiving more dynamic and complex visual inputs for a longer period of time (i.e. animation movies) than the mother (i.e. a black screen). Thus, if we compare intrabrain connectivity between conditions or between mothers and children, the systematic differences in visual stimulation would confound our results. However, in the present study, we are interested in comparing interbrain synchrony between conditions, instead of comparing intrabrain connectivity between conditions. Interbrain synchrony is supposedly induced by identical physical stimuli or communication goals. Because mothers and children were receiving different visual inputs, it is unlikely that the interbrain synchronization we observed was due to visual inputs.

### Interbrain coherence in the right inferior frontal gyrus

The right pars triangularis of the inferior frontal gyrus showed greater interbrain synchrony in the theta band across the modeling and imitation periods in both the mother and children. In addition, this area was also the common cortical hub observed across the modeling and imitation stages in the theta band in the mothers. In the children, the right pars triangularis of the inferior frontal gyrus appeared to be one of the cortical hubs during the modeling period in the theta band. In other words, this is the cortical hub that showed a high number of cross-brain connectivity and was commonly found in the theta band in the mothers and children during the modeling period.

In our intrabrain analysis (Supplementary Fig. S4), the right pars triangularis of the inferior frontal gyrus also appeared to be one of the cortical areas showing high intrabrain theta connectivity. That is, interbrain and intrabrain networks both recruited this right inferior frontal area in social interactions of turn-taking imitation. Using a single-person approach, the right inferior frontal area, in addition to the TPJ, is also considered one of the key regions in the human mentalizing network (Mar 2011; Redcay and Schilbach 2019). Common results in the intra- and inter-brain networks indicate the crucial roles of this cortical area in turn-taking verbal communications.

The verbal imitation task in our study entails social understanding and social interactions. Therefore, synchronized theta oscillations in the right parietal and frontal regions could reflect cognitive processes of mentalizing. It was proposed that the network of theory of mind involves bilateral TPJ and medial prefrontal cortex (Frith and Frith 2006; Mar 2011; Schurz et al. 2017). Other regions, such as the inferior frontal gyrus, posterior part of the superior temporal sulcus, and precuneus are also considered to be part of the network of theory of mind (Mar 2011; Redcay and Schilbach 2019). To perform the verbal imitation tasks in a socially interactive setting, a network of brain areas are engaged, including the inferior frontal and inferior parietal areas, both of which are involved in the human neural mirroring system (Iacoboni and Dapretto 2006; Hari and Kujala 2009; Hamilton 2013).

With a dual-fMRI setup and a joint attention task of mutual gaze, dyads showed synchronized activity in the right inferior frontal regions (Saito et al. 2010). Using a dual-fNIRS setup, enhanced interbrain synchronization was observed in the right inferior frontal areas during cooperative humming (Osaka et al. 2015). Similarly, when the interactive individuals were engaged in a cooperative button press task, the strategy of delayed responses resulted in higher interbrain synchrony in the right frontal regions compared to the strategy of instant responses (Tang et al. 2020). Results from these 2-brain studies reveal that the right inferior frontal area plays an important role in different aspects of social interactions.

In previous studies using dual-EEG recordings and source estimates, alpha activity in the right frontal regions were found to be related to understanding feelings and mental states of others (Babiloni et al. 2012). With a dual-EEG setup, increased interbrain synchronization was found in the theta- and beta-frequency ranges (Yun et al. 2012). Using source estimates, synchronized activity was observed between the right inferior frontal gyrus and the left postcentral gyrus as the fingertip movements of the 2 interactive individuals became synchronized. These findings support the role of synchronized alpha oscillations in joint attention and joint actions.

In addition, synchronized theta oscillations have been highlighted in the frontal electrodes in dual-EEG studies in social interactions. When a pair of guitarists played short melodies in unison, interbrain synchronization in the theta band increased at the central and frontal electrode sites when preparatory metronome beats were provided and when pairs of guitarists coordinated their play onsets (Lindenberger et al. 2009). In a later study (Sanger et al. 2012), guitar duets were assigned roles as a leader or a follower. Enhanced connection strengths in the delta and theta bands were also reported in the frontal electrodes across brains during periods of music coordination. Therefore, theta oscillations in interacting individuals are typically synchronized and enhanced during tasks requiring interactive and coordinated actions.

### Interbrain coherence in the left inferior frontal gyrus

The left pars opercularis of the inferior frontal gyrus showed increased theta interbrain synchrony across the modeling and imitations periods. Moreover, in the mothers, this area was also identified as one of the cortical hubs in the theta-frequency range during the imitation period, and in the alpha band during the modeling periods. In the children, the left pars opercularis of the inferior frontal gyrus was the cortical hub detected in the theta band across the modeling and imitation stages, and in the alpha-frequency range during the modeling stage. That is, in the theta band, the left opercular of the inferior frontal gyrus was the common cortical hub in the mothers and children during the imitation period. In the alpha band, this area was the common cortical hub found in the mothers and children during the modeling period.

To successfully perform our verbal interaction task, several speech-related processes are required, such as mapping auditory and motor representations attention, working memory, and attention. In our study, synchronized theta and alpha activity was observed in the inferior frontal areas. Hence, synchronized theta and alpha oscillations in the frontal regions could index these cognitive processes when turn-taking verbal imitation takes place.

Phonetic learning and speech imitation typically involve the left opercular part of the inferior frontal gyrus and inferior parietal regions (Golestani and Pallier 2007; Reiterer et al. 2011; Peschke et al. 2012). These regions are part of the dorsal stream of speech processing, supporting auditory–motor integration (Hickok and Poeppel 2004, 2007). Empirical evidence has shown the involvement of the dorsal stream during vocal imitation of speech using fMRI measurements (Reiterer et al. 2011; Garnier et al. 2013). The role of the inferior frontal region is also implicated in studies of social interactions as well as language learning (Li and Jeong 2020).

Interestingly, this dorsal pathway of speech overlaps partially with the ventral attention network, which includes the ventral inferior frontal region and the TPJ (Corbetta et al. 2008; Corbetta and Shulman 2011). Beyond the speech domain, the inferior frontal region is also involved during action observation and imitation (Kilner et al. 2009). Using dual-fNIRS recordings, increased activity was observed in the left pars opercularis of the inferior frontal gyrus during eye-to-eye contact compared to eye-to-picture gaze (Hirsch et al. 2017).

Overall, our findings are consistent with previous studies by showing that speech interaction requires interbrain synchrony across multiple frequency bands in multiple networks. In the present study, oscillatory activity synchronizes 2 brains in the theta and alpha bands. Synchronous oscillatory activity across communicating individuals was mainly detected in the parietal and frontal areas, which are included in the speech processing network, ventral attention network, and theory of mind networks. Accumulating evidence suggests that synchronized oscillations in multiple networks are involved to work collaboratively for accomplishing the interactive task at hand.

### Limitations and prospects

To the best of our knowledge, this is the first study investigating brain-to-brain synchrony during turn-based verbal interactions in mother–child dyads using dual-MEGs located in the same room. We also note some limitations.

Our sample size is relatively small. Future studies with larger sample sizes are needed to generalize and better understand links between interbrain synchrony and turn-taking behaviors using different interactive tasks and social contexts.

Our interactive and noninteraction condition differed in the degree of stimulus predictability. Behaviorally, top-down expectations of the rhythmic patterns in human speech are known to facilitate auditory detection and speech comprehension (Lawrance et al. 2014). The effects of stimulus predictability on brain activity have been demonstrated in several single-brain studies. For example, high predictability increased attentional gains and amplified brain activity in the posterior superior temporal area as shown in fMRI blood-oxygen-level-dependent (BOLD) responses (Dikker et al. 2014). More recently, a recent MEG study reported the effects of predictability on low-frequency (≤2 Hz) fluctuations in the auditory and sensorimotor areas (Koskinen et al. 2020). EEG studies also showed association of temporal predictability in auditory signals and delta oscillations (Herbst and Obleser 2019). Future studies could systematically manipulate stimulus predictability to probe its effects on interbrain synchrony.

The turn-taking verbal imitation paradigm used in our study is structured because the phrases and speech prosody were predetermined by the experimenters. This structured approach allows a moderate level of control. To be noted, this type of seminaturalistic (Nazneen et al. 2022) and quasi-realistic speech interaction is different from natural conversations. Verbal interaction can be studied using structured, semistructured, or unstructured approaches. For instance, structured dialogs were used with a dual-fNIRS setup to study teacher–student interactions (Holper et al. 2013). In semistructured verbal communications, topics were preselected by the experimenters and participants were allowed to converse on a specific topic for a limited period of time. With such semistructured dialogs, conversational pairs showed enhanced cross-brain synchronization during face-to-face communication (Jiang et al. 2012). During scenarios of problem solving or games that require verbal communication, the content of the conversations become unstructured but more naturalistic. In such cases, interbrain synchronization increased during such unstructured verbal communication compared to silent thinking (Nozawa et al. 2016). To gain full insight of the neural bases underlying verbal interactions, it is necessary to test interactive individuals in more unstructured and naturalistic contexts. Working toward a more naturalistic scenario, future studies could test mother–child interaction with unstructured dialogs.

Taking into account the above limitations, our findings remain important because brain-to-brain synchrony at the cortical level was found to be related to social interactions above and beyond the influence of shared external stimuli. In the present study, when cortico-vocal coherence was partialled out, brain-to-brain coherence was still observed during our turn-taking interactive task. Building on cortico-vocal coherence, we identified interbrain synchronization that could be associated with speech interactions. Moreover, the level of interbrain synchronization observed in our study was significantly stronger in the interactive compared to noninteraction condition. Based on our results, we speculate that interbrain synchronization could be first induced by the shared speech signal. Thus, the shared acoustic signals could play a role in enhancing shared attention on the interactive pairs of participants. Thereafter, coherent neural activity between 2 interactive individuals could be crucial and facilitative for successful verbal communication and learning, and may underpin the suggestion that language learning is “gated” by the social brain (Kuhl 2007). Findings from the partial coherence analysis call for future studies that record shared external stimuli in addition to simultaneous recordings of the 2 interactive brains when investigating interbrain synchronization. For example, in verbal communications, speech rhythm should be an additional measure to take into consideration.

## Conclusions

For the first time, cross-brain synchronization at the cortical level has been observed during mother–child interactions using a dual-MEG setup. Moreover, the design of our experiment isolates social interaction, as opposed to shared external sensory inputs, as the cause of the observed interbrain synchrony, therefore suggesting a potential neural marker of social verbal interaction. Our study highlights the advantages of dual-MEG setups to investigate 2-brain networks, which are manifested as synchronized interbrain oscillations in multiple frequency bands across multiple neural substrates.

Using a seminaturalistic turn-taking verbal interaction paradigm, our study suggests reciprocal social interaction between mother and child enhances brain-to-brain synchronization in the frontal and parietal regions. Our results also indicate that the 2 interacting brains were in synchrony with each other in terms of neural activity in multiple frequency bands during social interactions. The present study brings insight into our understanding of interbrain connectivity patterns during interactive behaviors in mother–child dyads. Our results also highlight the importance of verbal interaction in enhancing interpersonal neural synchrony. Moreover, our findings have implications for individuals with deficits in social communication, such as attention deficit/hyperactivity disorder or autism spectrum disorder. Simultaneous recordings of interactive brains in naturalistic settings open new doors for understanding neural processes of learning in different social contexts.

## Supplementary Material

Lin-supplementary_bhac330
