# Supplementary material for: Dual-MEG interbrain synchronization during turn-taking verbal interactions between mothers and children
Source: Cereb Cortex. 2022 Sep 20;33(7):4116–34. doi: 10.1093/cercor/bhac330 (PMC10068303; doi:10.1093/cercor/bhac330)
Supplement: Lin-supplementary_bhac330 [file lin-supplementary_bhac330.pdf]

Dual-MEG interbrain synchronization during turn-taking verbal interactions between mothers and children

Jo-Fu Lotus Lin[1,2,3], Toshiaki Imada[1,2], Andrew N Meltzoff[1], Hirotoshi Hiraishi[4], Takashi Ikeda[2], Tetsuya Takahashi[5], Chiaki Hasegawa[2], Yuko Yoshimura[2], Mitsuru Kikuchi[2], Masayuki Hirata[6], Yoshio Minabe[2], Minoru Asada[7], and Patricia K Kuhl[1]

Supplementary Information

Figure S1. Cortical regions showing significant interbrain synchrony using the original coherence measures. The original interbrain coherence was obtained before removing the contributions from auditory signals. On the inflated cortical surfaces, colors indicate significant differences between the socially interactive and non-interaction condition. Statistical results at the (a) theta, (b) alpha, and (c) low beta frequency bands are displayed.

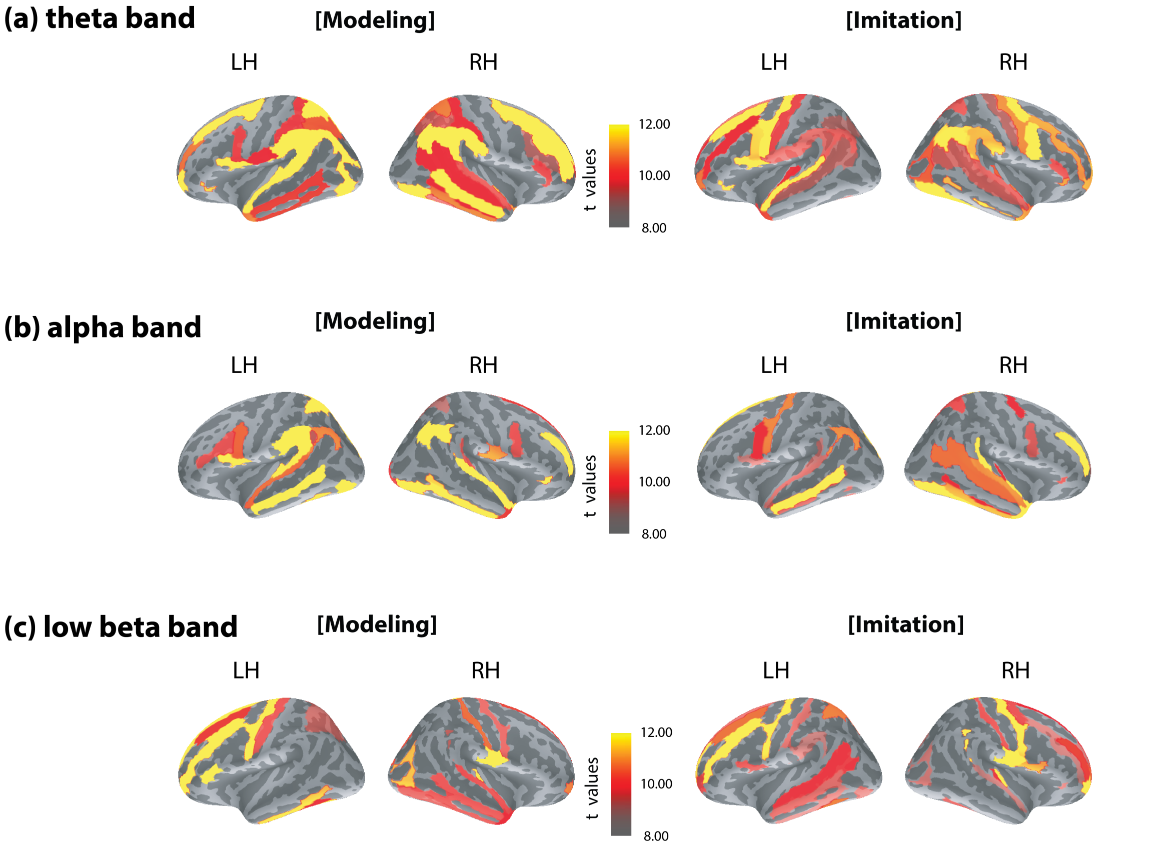

Figure S2. Differences observed between the original coherence and partial coherence measures. Statistical maps are displayed on the inflated brain surfaces. Colors denote brain areas showing significant differences between the original coherence (Figure S1) and partial coherence (Figure 3). Statistical results at the (a) theta, (b) alpha, and (c) low beta frequency bands are displayed.

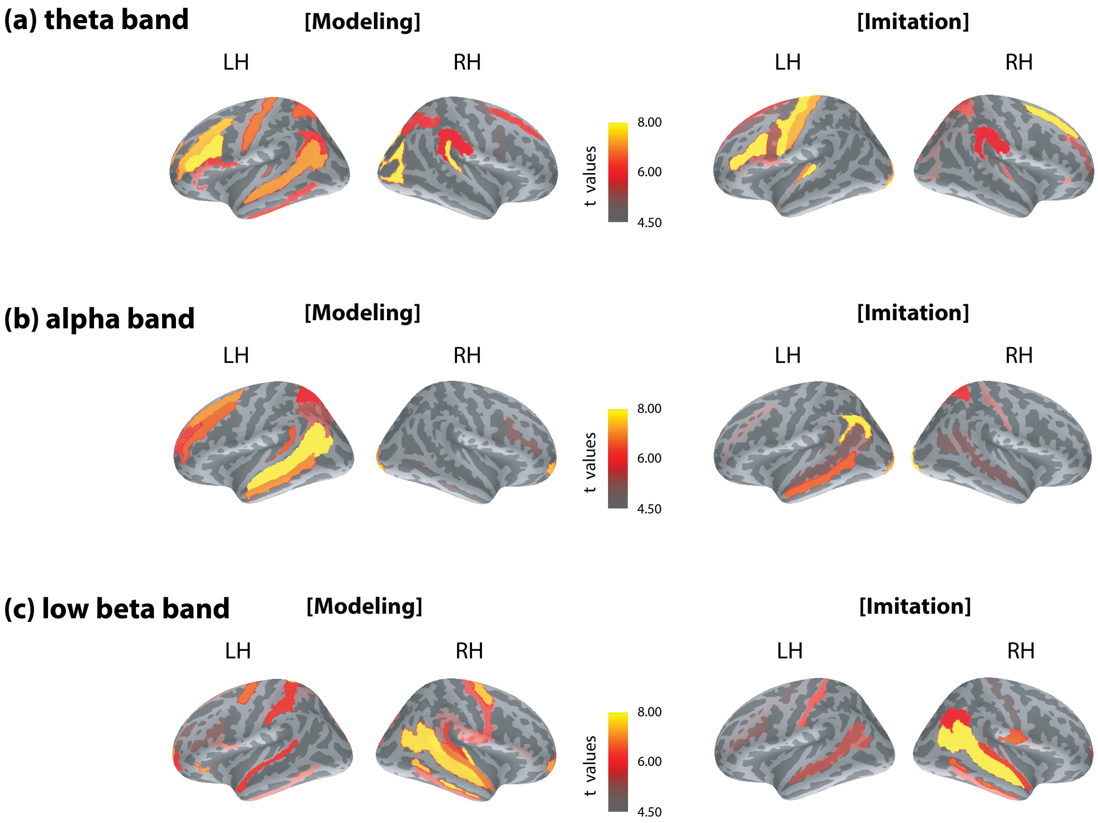

Figure S3. Interbrain synchrony detected during the socially interactive condition (Modeling and Imitation periods) and non-interactive condition (Pure Tone). Partial coherence values obtained in each condition were compared against zero coherence. Statistically significant interbrain connections at the (a) theta, (b) alpha, and (c) low beta frequency bands are displayed.

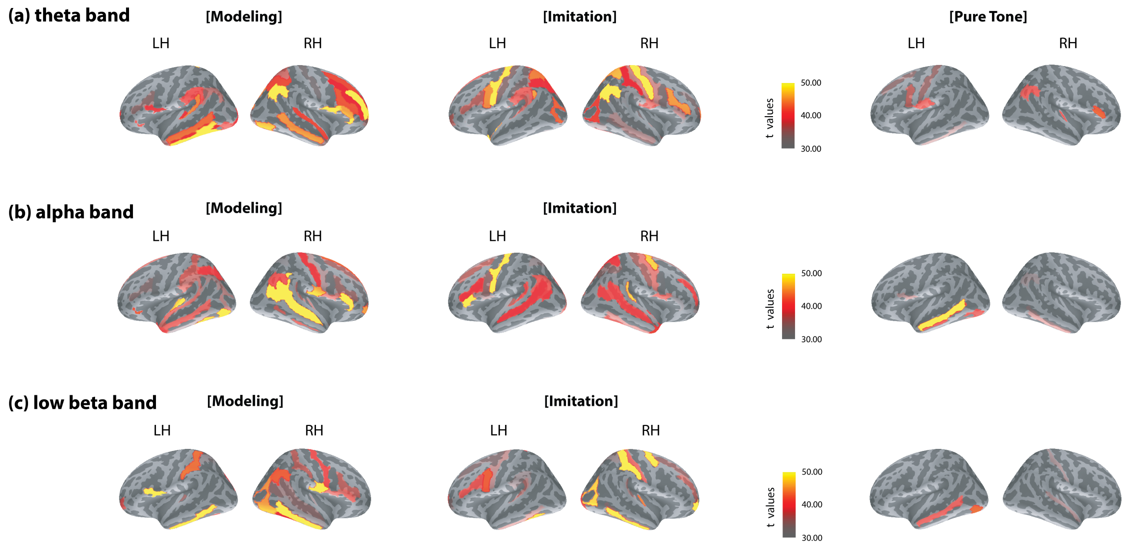

Figure S4. Cortical regions showing significant intrabrain connections in different frequency bands. ***Intrabrain connectivity: information hubs.***
To assess whether the interbrain hub regions overlap with hubs identified in the intrabrain analysis, we also calculated density measures based on intrabrain connectivity at the (a) theta, (b) alpha, and (c) low beta bands. As in the interbrain hub analysis, the degree measure was calculated by taking the ratio of significant intrabrain connections over the total number of possible intrabrain connections.

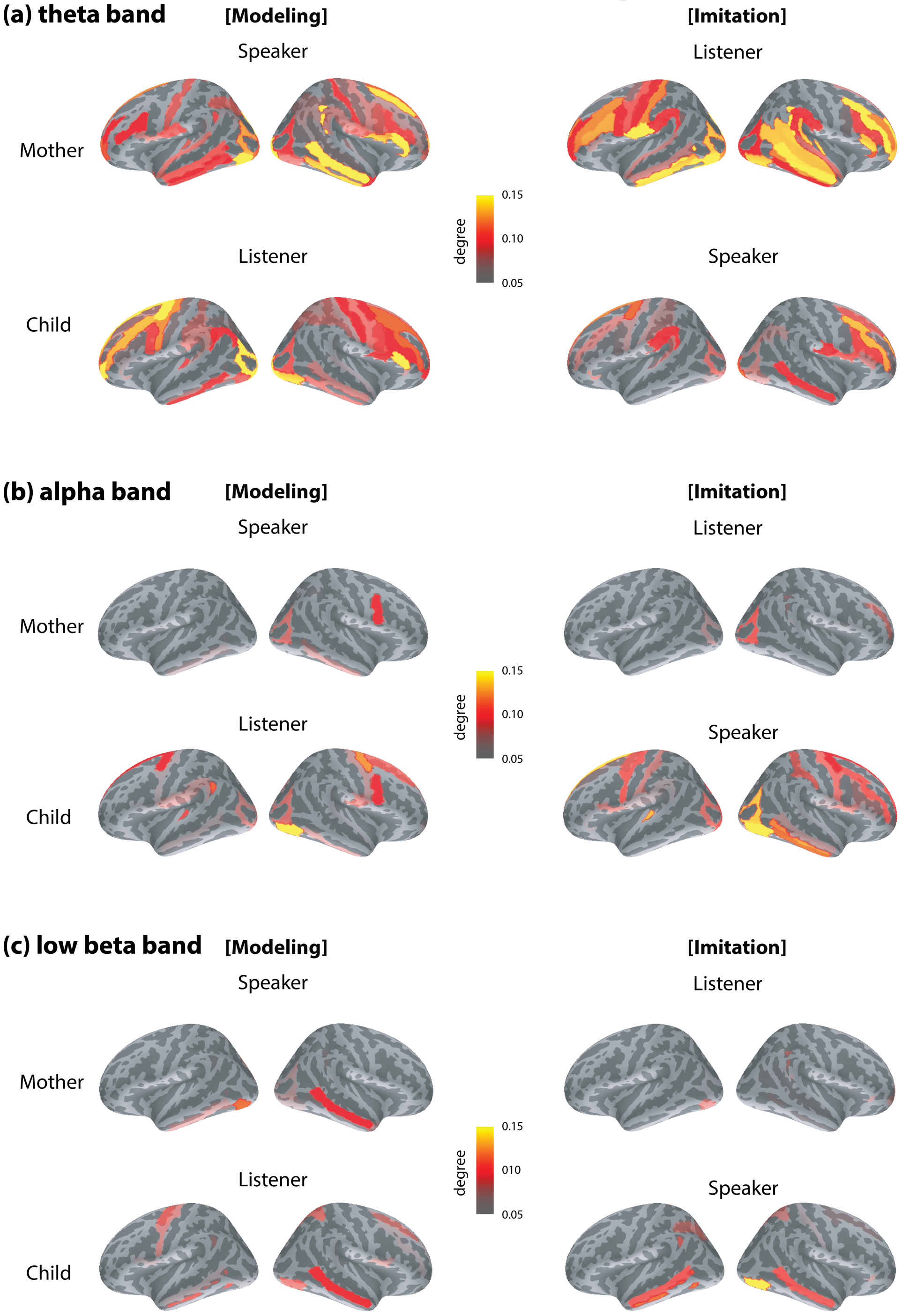

Table S1. Anatomical labels and numbering for the parcellated cortical regions. The short names of anatomical parcellations are based on Destrieux et al. (2010).

Mother's brain	Freesurfer anatomical label	Child's brain
M 0	LH G_frt_sup	C 0
M 1	LH G_frt_middle	C 1
M 2	LH G_frt_inf-Opercular	C 2
M 3	LH G_frt_inf-Orbital	C 3
M 4	LH G_frt_inf-Triangul	C 4
M 5	LH G_precentral	C 5
M 6	LH GS_frtomargin	C 6
M 7	LH GS_paracentral	C 7
M 8	LH GS_subcentral	C 8
M 9	LH GS_transv_frtopol	C 9
M 10	LH S_frt_sup	C 10
M 11	LH S_frt_middle	C 11
M 12	LH S_frt_inf	C 12
M 13	LH S_central	C 13
M 14	LH S_precentral-inf	C 14
M 15	LH S_precentral-sup	C 15
M 16	LH G_temp_sup-G_T_trns	C 16
M 17	LH G_temp_sup-Lateral	C 17
M 18	LH G_temp_sup-Plan_plr	C 18
M 19	LH G_temp_sup-Plan_tmp	C 19
M 20	LH G_temp_middle	C 20
M 21	LH G_temp_inf	C 21
M 22	LH Pole_temp	C 22
M 23	LH S_temp_sup	C 23
M 24	LH S_temp_transverse	C 24
M 25	LH S_temp_inf	C 25
M 26	LH G_postcentral	C 26
M 27	LH G_parietal_sup	C 27
M 28	LH G_pariet_inf-Angul	C 28
M 29	LH G_pariet_inf-Supram	C 29
M 30	LH G_precuneus	C 30
M 31	LH S_interm_prim-J	C 31
M 32	LH S_intraprt_P_trans	C 32
M 33	LH S_postcentral	C 33
M 34	LH S_subparietal	C 34

M 35	LH G_oc_sup	C 35
M 36	LH G_oc_middle	C 36
M 37	LH G_cuneus	C 37
M 38	LH G_oc-temp_lat-fusif	C 38
M 39	LH G_oc-temp_med-Ling	C 39
M 40	LH G_oc-temp_med-Parah	C 40
M 41	LH Pole_oc	C 41
M 42	LH GS_oc_inf	C 42
M 43	LH S_calcarine	C 43
M 44	RH G_frt_sup	C 44
M 45	RH G_frt_middle	C 45
M 46	RH G_frt_inf-Opercular	C 46
M 47	RH G_frt_inf-Orbital	C 47
M 48	RH G_frt_inf-Triangul	C 48
M 49	RH G_precentral	C 49
M 50	RH GS_frtomargin	C 50
M 51	RH GS_paracentral	C 51
M 52	RH GS_subcentral	C 52
M 53	RH GS_transv_frtopol	C 53
M 54	RH S_frt_sup	C 54
M 55	RH S_frt_middle	C 55
M 56	RH S_frt_inf	C 56
M 57	RH S_central	C 57
M 58	RH S_precentral-inf	C 58
M 59	RH S_precentral-sup	C 59
M 60	RH G_temp_sup-G_T_trns	C 60
M 61	RH G_temp_sup-Lateral	C 61
M 62	RH G_temp_sup-Plan_plr	C 62
M 63	RH G_temp_sup-Plan_tmp	C 63
M 64	RH G_temp_middle	C 64
M 65	RH G_temp_inf	C 65
M 66	RH Pole_temp	C 66
M 67	RH S_temp_sup	C 67
M 68	RH S_temp_transverse	C 68
M 69	RH S_temp_inf	C 69
M 70	RH G_postcentral	C 70
M 71	RH G_parietal_sup	C 71
M 72	RH G_pariet_inf-Angul	C 72
M 73	RH G_pariet_inf-Supram	C 73
M 74	RH G_precuneus	C 74
M 75	RH S_interm_prim-J	C 75
M 76	RH S_intraprt_P_trans	C 76
M 77	RH S_postcentral	C 77

M 78	RH S_subparietal	C 78
M 79	RH G_oc_sup	C 79
M 80	RH G_oc_middle	C 80
M 81	RH G_cuneus	C 81
M 82	RH G_oc-temp_lat-fusif	C 82
M 83	RH G_oc-temp_med-Ling	C 83
M 84	RH G_oc-temp_med-Parah	C 84
M 85	RH Pole_oc	C 85
M 86	RH GS_oc_inf	C 86
M 87	RH S_calcarine	C 87

Table S2. Interbrain synchrony of homologous areas in different task periods (Modeling, Imitation) and frequency bands (theta, alpha, beta). Statistical differences of partial coherence between the socially interactive and non-interactive condition are listed. Beta1 denotes the low beta frequency band. Cortical areas showing significant differences are also plotted on the inflated brain surface in Figure 3.

Brain regions		Period	Band	t values
LH	S_precentral-sup-part	Modeling	theta	14.53
LH	G_temp_sup-Plan_tempo	Modeling	theta	14.06
LH	G_front_inf-Opercular	Modeling	theta	13.93
LH	G_and_S_frontomargin	Modeling	theta	13.20
LH	S_interm_prim-Jensen	Modeling	theta	12.67
LH	G_temp_sup-Lateral	Modeling	theta	11.71
LH	S_parieto_occipital	Modeling	theta	11.66
LH	G_and_S_occipital_inf	Modeling	theta	11.49
LH	S_front_sup	Modeling	theta	11.47
LH	G_parietal_sup	Modeling	theta	11.30
LH	G_occipital_middle	Modeling	theta	10.94
LH	S_temporal_transverse	Modeling	theta	10.88
LH	G_pariet_inf-Angular	Modeling	theta	10.42
LH	Pole_temporal	Modeling	theta	9.70
LH	G_front_inf-Orbital	Modeling	theta	9.10
RH	S_front_sup	Modeling	theta	12.20
RH	S_precentral-sup-part	Modeling	theta	10.93
RH	G_front_middle	Modeling	theta	10.34
RH	G_pariet_inf-Angular	Modeling	theta	10.24
RH	G_front_inf-Triangul	Modeling	theta	10.19
RH	G_and_S_occipital_inf	Modeling	theta	9.92
RH	S_front_middle	Modeling	theta	9.71
RH	G_temporal_middle	Modeling	theta	9.48
RH	G_pariet_inf-Supramar	Modeling	theta	9.47
LH	G_precentral	Imitation	theta	13.50
LH	S_precentral-inf-part	Imitation	theta	10.58
LH	G_front_inf-Opercular	Imitation	theta	10.23
LH	S_front_sup	Imitation	theta	10.00
LH	G_and_S_frontomargin	Imitation	theta	9.65
RH	S_precentral-inf-part	Imitation	theta	14.49
RH	S_precentral-sup-part	Imitation	theta	11.40
RH	G_pariet_inf-Angular	Imitation	theta	11.12
RH	S_temporal_transverse	Imitation	theta	10.29
RH	G_front_inf-Triangul	Imitation	theta	9.62
RH	G_temp_sup-Plan_polar	Imitation	theta	9.53
LH	S_precentral-inf-part	Modeling	alpha	12.48
LH	G_front_inf-Opercular	Modeling	alpha	12.23
LH	G_pariet_inf-Supramar	Modeling	alpha	11.98

LH	G_temporal_middle	Modeling	alpha	11.64
LH	G_and_S_occipital_inf	Modeling	alpha	11.48
LH	G_temp_sup-Plan_tempo	Modeling	alpha	11.20
LH	S_temporal_transverse	Modeling	alpha	10.35
RH	G_temp_sup-Lateral	Modeling	alpha	12.96
RH	G_pariet_inf-Angular	Modeling	alpha	12.74
RH	G_temp_sup-Plan_polar	Modeling	alpha	12.67
RH	G_and_S_occipital_inf	Modeling	alpha	11.85
RH	G_front_inf-Orbital	Modeling	alpha	11.55
RH	S_front_middle	Modeling	alpha	10.72
RH	G_front_sup	Modeling	alpha	9.35
LH	S_precentral-inf-part	Imitation	alpha	10.46
LH	G_temporal_middle	Imitation	alpha	9.74
LH	G_front_sup	Imitation	alpha	9.46
LH	G_front_inf-Opercular	Imitation	alpha	9.01
RH	G_temp_sup-Plan_tempo	Imitation	alpha	14.72
RH	G_temp_sup-Plan_polar	Imitation	alpha	14.16
RH	G_and_S_paracentral	Imitation	alpha	13.31
RH	G_and_S_occipital_inf	Imitation	alpha	10.62
RH	S_calcarine	Imitation	alpha	10.19
RH	S_oc-temp_lat	Imitation	alpha	10.15
RH	G_temporal_inf	Imitation	alpha	9.40
RH	Pole_temporal	Imitation	alpha	9.17
RH	G_front_inf-Orbital	Imitation	alpha	9.11
LH	G_front_inf-Triangul	Modeling	beta1	19.95
LH	G_front_middle	Modeling	beta1	12.98
LH	G_and_S_frontomargin	Modeling	beta1	10.48
LH	G_oc-temp_lat-fusifor	Modeling	beta1	9.90
LH	G_precentral	Modeling	beta1	9.35
RH	G_and_S_subcentral	Modeling	beta1	11.73
RH	G_and_S_paracentral	Modeling	beta1	10.66
RH	G_cuneus	Modeling	beta1	9.40
RH	G_occipital_sup	Modeling	beta1	9.27
RH	G_occipital_middle	Modeling	beta1	9.23
RH	G_precuneus	Modeling	beta1	9.06
LH	G_oc-temp_med-Lingual	Imitation	beta1	9.93
LH	S_front_sup	Imitation	beta1	9.33
RH	G_precuneus	Imitation	beta1	12.48
RH	S_interm_prim-Jensen	Imitation	beta1	11.51
RH	G_and_S_frontomargin	Imitation	beta1	10.39
RH	G_and_S_paracentral	Imitation	beta1	10.14
RH	G_cuneus	Imitation	beta1	9.93
RH	G_and_S_subcentral	Imitation	beta1	9.86
RH	G_front_inf-Opercular	Imitation	beta1	9.77

Table S3. Interbrain connectivity in non-homogenous cortical areas in different task periods (Modeling, Imitation) and frequency bands (theta, alpha, low beta). Beta1 indicates the low beta frequency band. Interbrain connections are visualized in circular plots in Figure 4.

ROI pairs Mother-Child	Mother Brain		Child Brain		Period	Band	t values
**Left Hemisphere**							
M31-C17	MLH	S_interm_prim-J	CLH	G_temp_sup-Lateral	Modeling	theta	25.94
M2-C14	MLH	G_frt_inf-Opercular	CLH	S_precentral-inf	Modeling	theta	23.01
M0-C19	MLH	G_frt_sup	CLH	G_temp_sup-Plan_tmp	Modeling	theta	19.14
M27-C28	MLH	G_parietal_sup	CLH	G_pariet_inf-Angul	Modeling	theta	19.08
M13-C4	MLH	S_central	CLH	G_frt_inf-Triangul	Modeling	theta	19.08
M21-C0	MLH	G_temp_inf	CLH	G_frt_sup	Modeling	theta	18.18
**Right Hemisphere**							
M67-C49	MRH	S_temp_sup	CRH	G_precentral	Modeling	theta	22.64
M55-C62	MRH	S_frt_middle	CRH	G_temp_sup-Plan_plr	Modeling	theta	20.62
M64-C45	MRH	G_temp_middle	CRH	G_frt_middle	Modeling	theta	20.50
M63-C48	MRH	G_temp_sup-Plan_tmp	CRH	G_frt_inf-Triangul	Modeling	theta	17.26
**Across Hemispheres**							
M38-C47	MLH	G_oc-temp_lat-fusif	CRH	G_frt_inf-Orbital	Modeling	theta	28.58
M62-C25	MRH	G_temp_sup-Plan_plr	CLH	S_temp_inf	Modeling	theta	22.31
M26-C85	MLH	G_postcentral	CRH	Pole_oc	Modeling	theta	20.27
M4-C68	MLH	G_frt_inf-Triangul	CRH	S_temp_transverse	Modeling	theta	17.43
**Left Hemisphere**							
M4-C11	MLH	G_frt_inf-Triangul	CLH	S_frt_middle	Imitation	theta	28.98
M16-C29	MLH	G_temp_sup-G_T_trns	CLH	G_pariet_inf-Supram	Imitation	theta	18.75
M20-C7	MLH	G_temp_middle	CLH	GS_paracentral			18.63
**Right Hemisphere**							
M68-C58	MRH	S_temp_transverse	CRH	S_precentral-inf	Imitation	theta	22.70
M82-C54	MRH	G_oc-temp_lat-fusif	CRH	S_frt_sup	Imitation	theta	20.71
M87-C54	MRH	S_calcarine	CRH	S_frt_sup	Imitation	theta	20.29
M46-C68	MRH	G_frt_inf-Opercular	CRH	S_temp_transverse	Imitation	theta	18.79
M75-C70	MRH	S_interm_prim-J	CRH	G_postcentral	Imitation	theta	18.62
M61-C58	MRH	G_temp_sup-Lateral	CRH	S_precentral-inf	Imitation	theta	18.05
**Across Hemispheres**							
M52-C6	MRH	GS_subcentral	CLH	GS_frtomargin	Imitation	theta	30.64
M35-C54	MLH	G_oc_sup	CRH	S_frt_sup	Imitation	theta	22.19
M17-C60	MLH	G_temp_sup-Lateral	CRH	G_temp_sup-G_T_trns	Imitation	theta	20.94
M52-C35	MRH	GS_subcentral	CLH	G_oc_sup	Imitation	theta	20.09
M41-C81	MLH	Pole_oc	CRH	G_cuneus	Imitation	theta	19.91
M29-C75	MLH	G_pariet_inf-Supram	CRH	S_interm_prim-J	Imitation	theta	18.26

**Left Hemisphere**							
M1-C10	CLH	G_frt_middle	CLH	S_frt_sup	Modeling	alpha	21.81
M24-C3	MLH	S_temp_transverse	CLH	G_frt_inf-Orbital	Modeling	alpha	20.62
M12-C35	MLH	S_frt_inf	CLH	G_oc_sup	Modeling	alpha	18.82
M2-C14	MLH	G_frt_inf-Opercular	CLH	S_precentral-inf	Modeling	alpha	18.47
M37-C2	MLH	G_cuneus	CLH	G_frt_inf-Opercular	Modeling	alpha	18.45
M32-C12	MLH	S_intraprt_P_trans	CLH	S_frt_inf	Modeling	alpha	18.37
M18-C33	MLH	G_temp_sup-Plan_plr	CLH	S_postcentral	Modeling	alpha	17.68
**Right Hemisphere**							
M71-C69	MRH	G_parietal_sup	CRH	S_temp_inf	Modeling	alpha	18.22
M46-C63	MRH	G_frt_inf-Opercular	CRH	G_temp_sup-Plan_tmp	Modeling	alpha	17.79
M84-C65	MRH	G_oc-temp_med-Parah	CRH	G_temp_inf	Modeling	alpha	17.19
**Across Hemispheres**							
M3-C63	MLH	G_frt_inf-Orbital	CRH	G_temp_sup-Plan_tmp	Modeling	alpha	20.99
M66-C2	MRH	Pole_temp	CLH	G_frt_inf-Opercular	Modeling	alpha	19.03
M41-C47	MLH	Pole_oc	CRH	G_frt_inf-Orbital	Modeling	alpha	18.18
M64-C7	MRH	G_temp_middle	CLH	GS_paracentral	Modeling	alpha	17.98
M65-C18	MRH	G_temp_inf	CLH	G_temp_sup-Plan_plr	Modeling	alpha	17.39
M2-C62	MLH	G_frt_inf-Opercular	CRH	G_temp_sup-Plan_plr	Modeling	alpha	17.33

**Left Hemisphere**							
M21-C32	MLH	G_temp_inf	CLH	S_intraprt_P_trans	Imitation	alpha	19.21
**Right Hemisphere**							
M55-C81	MRH	S_frt_middle	CRH	G_cuneus	Imitation	alpha	40.38
M67-C77	MRH	S_temp_sup	CRH	S_postcentral	Imitation	alpha	19.18
M64-C59	MRH	G_temp_middle	CRH	S_precentral-sup	Imitation	alpha	18.29
**Across Hemispheres**							
M66-C0	MRH	Pole_temp	CLH	G_frt_sup	Imitation	alpha	19.44
M3-C66	MLH	G_frt_inf-Orbital	CRH	Pole_temp	Imitation	alpha	19.12
M7-C75	MLH	GS_paracentral	CRH	S_interm_prim-J	Imitation	alpha	18.54
M53-C24	MRH	GS_transv_frtopol	CLH	S_temp_transverse	Imitation	alpha	17.54
M66-C32	MRH	Pole_temp	CLH	S_intraprt_P_trans	Imitation	alpha	17.09
M46-C7	MRH	G_frt_inf-Opercular	CLH	GS_paracentral	Imitation	alpha	17.08

**Left Hemisphere**							
M4-C4	MLH	G_frt_inf-Triangul	CLH	G_frt_inf-Triangul	Modeling	beta1	19.95
M42-C14	MLH	GS_oc_inf	CLH	S_precentral-inf	Modeling	beta1	17.78
**Right Hemisphere**							
M82-C59	MRH	G_oc-temp_lat-fusif	CRH	S_precentral-sup	Modeling	beta1	19.61
M55-C74	MRH	S_frt_middle	CRH	G_precuneus	Modeling	beta1	18.62
M60-C52	MRH	G_temp_sup-G_T_trns	CRH	GS_subcentral	Modeling	beta1	17.97
**Across Hemispheres**							
M77-C24	MRH	S_postcentral	CLH	S_temp_transverse	Modeling	beta1	20.59
M69-C38	MRH	S_temp_inf	CLH	G_oc-temp_lat-fusif	Modeling	beta1	19.60
M5-C80	MLH	G_precentral	CRH	G_oc_middle	Modeling	beta1	19.12
M45-C33	MRH	G_frt_middle	CLH	S_postcentral	Modeling	beta1	18.88
M52-C2	MRH	GS_subcentral	CLH	G_frt_inf-Opercular	Modeling	beta1	18.12

M87-C13	MRH	S_calcarine	CLH	S_central	Modeling	beta1	17.91
M57-C9	MRH	S_central	CLH	GS_transv_frtopol	Modeling	beta1	17.59
**Left Hemisphere**							
M26-C22	MLH	G_postcentral	CLH	Pole_temp	Imitation	beta1	21.47
M30-C25	MLH	G_precuneus	CLH	S_temp_inf	Imitation	beta1	19.48
M15-C16	MLH	S_precentral-sup	CLH	G_temp_sup-G_T_trns	Imitation	beta1	18.74
M26-C16	MLH	G_postcentral	CLH	G_temp_sup-G_T_trns	Imitation	beta1	17.24
M27-C16	MLH	G_parietal_sup	CLH	G_temp_sup-G_T_trns	Imitation	beta1	17.22
**Right Hemisphere**							
M46-C54	MRH	G_frt_inf-Opercular	CRH	S_frt_sup	Imitation	beta1	23.12
M83-C58	MRH	G_oc-temp_med-Ling	CRH	S_precentral-inf	Imitation	beta1	20.50
M5-C30	MLH	G_precentral	CLH	G_precuneus	Imitation	beta1	17.47
**Across Hemispheres**							
M48-C24	MRH	G_frt_inf-Triangul	CLH	S_temp_transverse	Imitation	beta1	18.74
M13-C45	MLH	S_central	CRH	G_frt_middle	Imitation	beta1	18.55
M10-C50	MLH	S_frt_sup	CRH	GS_frtomargin	Imitation	beta1	17.56
M47-C36	MRH	G_frt_inf-Orbital	CLH	G_oc_middle	Imitation	beta1	17.50
M61-C39	MRH	G_temp_sup-Lateral	CLH	G_oc-temp_med-Ling	Imitation	beta1	17.35
M14-C45	MLH	S_precentral-inf	CRH	G_frt_middle	Imitation	beta1	17.12

Table S4. Brain regions identified as cortical hubs in different task periods (Modeling, Imitation) and frequency bands (theta, alpha, low beta). Beta1 denotes the low beta frequency band. Cortical hubs are displayed on the inflated brain surface in Figure 5.

**Hubs (Brain areas)**			**Period**	**Band**	**density**
MLH	G_and_S_frontomargin	Mother	Modeling	theta	0.26
MLH	G_and_S_occipital_inf	Mother	Modeling	theta	0.25
MLH	G_and_S_paracentral	Mother	Modeling	theta	0.25
MLH	S_postcentral	Mother	Modeling	theta	0.24
MLH	S_interm_prim-Jensen	Mother	Modeling	theta	0.22
MLH	G_temp_sup-Lateral	Mother	Modeling	theta	0.22
MLH	Pole_temporal	Mother	Modeling	theta	0.20
MLH	S_temporal_transverse	Mother	Modeling	theta	0.20
MLH	G_front_inf-Orbital	Mother	Modeling	theta	0.20
MLH	G_front_sup	Mother	Modeling	theta	0.20
MLH	S_front_inf	Mother	Modeling	theta	0.18
MLH	G_and_S_subcentral	Mother	Modeling	theta	0.18
MLH	S_temporal_inf	Mother	Modeling	theta	0.17
MLH	S_temporal_sup	Mother	Modeling	theta	0.17
MRH	G_front_inf-Triangul	Mother	Modeling	theta	0.35
MRH	G_front_inf-Orbital	Mother	Modeling	theta	0.28
MRH	Pole_occipital	Mother	Modeling	theta	0.25
MRH	S_front_inf	Mother	Modeling	theta	0.24
MRH	S_precentral-inf-part	Mother	Modeling	theta	0.24
MRH	G_precentral	Mother	Modeling	theta	0.24
MRH	G_and_S_transv_frontopol	Mother	Modeling	theta	0.23
MRH	G_pariet_inf-Angular	Mother	Modeling	theta	0.23
MRH	G_and_S_frontomargin	Mother	Modeling	theta	0.21
MRH	G_front_sup	Mother	Modeling	theta	0.21
MRH	G_temporal_middle	Mother	Modeling	theta	0.20
MRH	G_occipital_middle	Mother	Modeling	theta	0.19
MRH	G_temp_sup-Plan_polar	Mother	Modeling	theta	0.19
MRH	G_front_inf-Opercular	Mother	Modeling	theta	0.18
MRH	G_temp_sup-G_T_transv	Mother	Modeling	theta	0.18
CLH	G_front_inf-Opercular	Child	Modeling	theta	0.24
CLH	G_temporal_inf	Child	Modeling	theta	0.23
CLH	G_pariet_inf-Angular	Child	Modeling	theta	0.22
CLH	S_temporal_inf	Child	Modeling	theta	0.22
CLH	G_front_sup	Child	Modeling	theta	0.20
CLH	G_oc-temp_med-Parahip	Child	Modeling	theta	0.20
CLH	G_front_middle	Child	Modeling	theta	0.20
CLH	G_parietal_sup	Child	Modeling	theta	0.18
CLH	S_front_inf	Child	Modeling	theta	0.18
CLH	S_temporal_transverse	Child	Modeling	theta	0.18
CLH	G_front_inf-Triangul	Child	Modeling	theta	0.17
CLH	G_temporal_middle	Child	Modeling	theta	0.17

CLH	S_interm_prim-Jensen	Child	Modeling	theta	0.17
CLH	S_intrapariet_and_P_trans	Child	Modeling	theta	0.17
CLH	S_precentral-sup-part	Child	Modeling	theta	0.16
CRH	G_temporal_middle	Child	Modeling	theta	0.24
CRH	G_pariet_inf-Angular	Child	Modeling	theta	0.22
CRH	G_temporal_inf	Child	Modeling	theta	0.22
CRH	S_temporal_inf	Child	Modeling	theta	0.22
CRH	G_front_inf-Orbital	Child	Modeling	theta	0.22
CRH	S_temporal_sup	Child	Modeling	theta	0.20
CRH	S_precentral-inf-part	Child	Modeling	theta	0.19
CRH	G_and_S_subcentral	Child	Modeling	theta	0.18
CRH	S_interm_prim-Jensen	Child	Modeling	theta	0.18
CRH	S_temporal_transverse	Child	Modeling	theta	0.18
CRH	G_and_S_frontomargin	Child	Modeling	theta	0.18
CRH	G_and_S_occipital_inf	Child	Modeling	theta	0.18
CRH	G_front_inf-Triangul	Child	Modeling	theta	0.18
CRH	G_temp_sup-Plan_polar	Child	Modeling	theta	0.18
CRH	G_occipital_middle	Child	Modeling	theta	0.17
CRH	S_front_middle	Child	Modeling	theta	0.17
CRH	G_front_middle	Child	Modeling	theta	0.16
CRH	G_parietal_sup	Child	Modeling	theta	0.16
CRH	S_front_sup	Child	Modeling	theta	0.16
MLH	G_and_S_frontomargin	Mother	Imitation	theta	0.21
MLH	S_front_inf	Mother	Imitation	theta	0.21
MLH	G_and_S_subcentral	Mother	Imitation	theta	0.18
MLH	G_front_inf-Opercular	Mother	Imitation	theta	0.18
MLH	G_front_inf-Orbital	Mother	Imitation	theta	0.18
MLH	S_central	Mother	Imitation	theta	0.18
MLH	G_and_S_occipital_inf	Mother	Imitation	theta	0.17
MLH	G_temp_sup-G_T_transv	Mother	Imitation	theta	0.16
MRH	G_front_inf-Triangul	Mother	Imitation	theta	0.36
MRH	G_and_S_paracentral	Mother	Imitation	theta	0.34
MRH	S_precentral-inf-part	Mother	Imitation	theta	0.30
MRH	G_pariet_inf-Angular	Mother	Imitation	theta	0.23
MRH	S_front_sup	Mother	Imitation	theta	0.22
MRH	G_precentral	Mother	Imitation	theta	0.22
MRH	Pole_occipital	Mother	Imitation	theta	0.22
MRH	S_temporal_transverse	Mother	Imitation	theta	0.21
MRH	G_temp_sup-Plan_polar	Mother	Imitation	theta	0.19
MRH	G_front_sup	Mother	Imitation	theta	0.18
MRH	S_precentral-sup-part	Mother	Imitation	theta	0.18
CLH	G_pariet_inf-Angular	Child	Imitation	theta	0.24
CLH	G_temporal_middle	Child	Imitation	theta	0.22
CLH	S_temporal_transverse	Child	Imitation	theta	0.20
CLH	G_temp_sup-Plan_tempo	Child	Imitation	theta	0.18
CLH	S_temporal_inf	Child	Imitation	theta	0.18
CLH	S_front_sup	Child	Imitation	theta	0.17

CLH	S_intrapariet_and_P_trans	Child	Imitation	theta	0.17
CLH	G_front_inf-Opercular	Child	Imitation	theta	0.16
CLH	G_pariet_inf-Supramar	Child	Imitation	theta	0.16
CLH	G_temp_sup-G_T_transv	Child	Imitation	theta	0.16
CRH	G_temporal_middle	Child	Imitation	theta	0.24
CRH	S_temporal_transverse	Child	Imitation	theta	0.23
CRH	G_and_S_subcentral	Child	Imitation	theta	0.20
CRH	G_and_S_occipital_inf	Child	Imitation	theta	0.16
**Hubs (Brain areas)**			**Period**	**Band**	**density**
MLH	G_front_sup	Mother	Modeling	alpha	0.34
MLH	G_front_inf-Opercular	Mother	Modeling	alpha	0.27
MLH	S_temporal_transverse	Mother	Modeling	alpha	0.26
MLH	S_precentral-inf-part	Mother	Modeling	alpha	0.25
MLH	S_precentral-sup-part	Mother	Modeling	alpha	0.20
MLH	G_pariet_inf-Supramar	Mother	Modeling	alpha	0.18
MLH	G_front_inf-Orbital	Mother	Modeling	alpha	0.18
MLH	S_temporal_sup	Mother	Modeling	alpha	0.18
MLH	G_temp_sup-Lateral	Mother	Modeling	alpha	0.16
MLH	S_postcentral	Mother	Modeling	alpha	0.16
MRH	G_precentral	Mother	Modeling	alpha	0.20
MRH	G_temporal_inf	Mother	Modeling	alpha	0.20
MRH	G_temp_sup-Plan_tempo	Mother	Modeling	alpha	0.16
CLH	G_front_inf-Orbital	Child	Modeling	alpha	0.22
CLH	G_front_inf-Opercular	Child	Modeling	alpha	0.16
CLH	G_front_middle	Child	Modeling	alpha	0.16
CRH	G_front_inf-Orbital	Child	Modeling	alpha	0.18
CRH	G_oc-temp_med-Parahip	Child	Modeling	alpha	0.18
CRH	G_temporal_inf	Child	Modeling	alpha	0.16
CRH	Pole_temporal	Child	Modeling	alpha	0.16
CRH	S_front_middle	Child	Modeling	alpha	0.16
MLH	S_temporal_transverse	Mother	Imitation	alpha	0.22
MLH	G_and_S_subcentral	Mother	Imitation	alpha	0.18
MLH	G_front_sup	Mother	Imitation	alpha	0.17
MRH	G_occipital_middle	Mother	Imitation	alpha	0.29
MRH	S_interm_prim-Jensen	Mother	Imitation	alpha	0.25
MRH	G_temp_sup-Plan_polar	Mother	Imitation	alpha	0.18
CRH	Pole_temporal	Child	Imitation	alpha	0.21
CRH	G_front_inf-Orbital	Child	Imitation	alpha	0.16
CRH	S_front_middle	Child	Imitation	alpha	0.16
**Hubs (Brain areas)**			**Period**	**Band**	**density**
MLH	S_precentral-inf-part	Mother	Modeling	beta1	0.24
MLH	S_front_inf	Mother	Modeling	beta1	0.21
MLH	G_front_middle	Mother	Modeling	beta1	0.16
MRH	G_occipital_middle	Mother	Modeling	beta1	0.18

CRH	S_precentral-inf-part	Child	Modeling	beta1	0.18
MLH	G_and_S_paracentral	Mother	Imitation	beta1	0.20
MLH	S_postcentral	Mother	Imitation	beta1	0.19
MLH	G_and_S_subcentral	Mother	Imitation	beta1	0.17
MLH	G_pariet_inf-Angular	Mother	Imitation	beta1	0.17
MLH	G_temp_sup-G_T_transv	Mother	Imitation	beta1	0.16
MRH	S_precentral-sup-part	Mother	Imitation	beta1	0.16
CLH	G_postcentral	Child	Imitation	beta1	0.24
CLH	S_front_sup	Child	Imitation	beta1	0.16
CRH	S_front_middle	Child	Imitation	beta1	0.18
CRH	S_interm_prim-Jensen	Child	Imitation	beta1	0.16
